# Explosive-driven double-blast exposure: molecular, histopathological, and behavioral consequences

**DOI:** 10.1038/s41598-020-74296-2

**Published:** 2020-10-15

**Authors:** Erin K. Murphy, Diego Iacono, Hongna Pan, Jamie B. Grimes, Steven Parks, Sorana Raiciulescu, Fabio Leonessa, Daniel P. Perl

**Affiliations:** 1 DoD/USU Brain Tissue Repository and Neuropathology Core, Uniformed Services University (USU), Bethesda, MD USA; 2 The Henry M. Jackson Foundation (HJF) for the Advancement of Military Medicine, Bethesda, MD USA; 3 Department of Neurology, F. Edward Hébert School of Medicine, Uniformed Services University (USU), 4301 Jones Bridge Rd, Bethesda, MD 20814 USA; 4 Department Pathology, F. Edward Hébert School of Medicine, Uniformed Services University (USU), 4301 Jones Bridge Rd, Bethesda, MD 20814 USA; 5 Neurodegenerative Clinics, National Institute of Neurological Disorders and Stroke (NINDS), NIH, Bethesda, MD USA; 6 ORA, Inc., 315 Baldwin Ave, Marion, NC 28752 USA; 7 Department of Preventive Medicine and Biostatistics, Biostatistics Consulting Center, F. Edward Hébert School of Medicine, Uniformed Services University (USU), 4301 Jones Bridge Rd, Bethesda, MD 20814 USA; 8 Department of Biochemistry, Uniformed Services University (USU), 4301 Jones Bridge Rd, Bethesda, MD 20814 USA

**Keywords:** Neurology, Brain injuries

## Abstract

Traumatic brain injury generated by blast may induce long-term neurological and psychiatric sequelae. We aimed to identify molecular, histopathological, and behavioral changes in rats 2 weeks after explosive-driven double-blast exposure. Rats received two 30-psi (~ 207-kPa) blasts 24 h apart or were handled identically without blast. All rats were behaviorally assessed over 2 weeks. At Day 15, rats were euthanized, and brains removed. Brains were dissected into frontal cortex, hippocampus, cerebellum, and brainstem. Western blotting was performed to measure levels of total-Tau, phosphorylated-Tau (pTau), amyloid precursor protein (APP), GFAP, Iba1, αII-spectrin, and spectrin breakdown products (SBDP). Kinases and phosphatases, correlated with tau phosphorylation were also measured. Immunohistochemistry for pTau, APP, GFAP, and Iba1 was performed. pTau protein level was greater in the hippocampus, cerebellum, and brainstem and APP protein level was greater in cerebellum of blast vs control rats (*p* < 0.05). GFAP, Iba1, αII-spectrin, and SBDP remained unchanged. No immunohistochemical or neurobehavioral changes were observed. The dissociation between increased pTau and APP in different regions in the absence of neurobehavioral changes 2 weeks after double blast exposure is a relevant finding, consistent with human data showing that battlefield blasts might be associated with molecular changes before signs of neurological and psychiatric disorders manifest.

## Introduction

Exposure to explosive blasts, especially from improvised explosive devices (IEDs), has been a relatively common event for military personnel deployed in Afghanistan and Iraq during the last 20 years of conflicts^1–3^. Acute blast exposures are often associated with subconcussion, concussion, and traumatic brain injury (TBI) events and are suspected to also initiate long-term neuropathological changes that might culminate in persistent alterations of the normal functioning of the brain and later clinical appearance of a wide spectrum of neurological and psychiatric disorders (from vestibular disorders to suicidal ideation)^4^. While explosive events can cause injury by several mechanisms (e.g. acceleration/deceleration, sudden impact, penetration), the true impact of blast’s most specific mechanism of injury (“*primary effect*”)—the one mediated by transmission of blast waves through the body, cavum organs (e.g., intestine, lungs) and skull on the brain’s functional and structural integrity—is still not very well understood^3,5–8^.

Although the impact of repeated blast exposures is of great concern, it has been addressed primarily in preclinical studies using non-explosive-driven blast generated pneumatically in blast tubes reducing the degree of real-life conditions^9,10^. One recent study^11^ investigated repeated blast using the well characterized Karolinska Institute model based on the more realistic Clemedson tube^12^, which utilizes detonated explosives in comparison to a pneumatically gas driven blast tube. Importantly, this study highlights differences in the outcome measures between pneumatic (shock tubes) and explosive driven repeated blast exposure. Our study also utilizes an explosive driven blast tube similar to the Karolinska model. There have also been a limited number of studies published in rodents as well as larger animals (e.g. primates and swine) using open field settings with exposure to detonated explosives which truly begin to reproduce the real life settings of military and civilian blast exposures^10,13–18^. While open field environments are the most realistic, there are difficulties in carrying out open field studies including but not limited to: large amounts of required explosives and securing an accessible location where such studies can occur^10^.

Given the high likelihood that Service Members (and civilians) currently in war theaters are exposed to multiple blast events of various modalities (e.g. multiple simultaneous IEDs, accumulated low level exposures from heavy weapons, two mines exploding simultaneously, etc*.)*, it is urgent that the effects of multiple blast exposures be experimentally explored in order to shed light on their possible neurobehavioral, molecular, and possible neurohistopathological consequences. It is also of utmost importance that we try to utilize animal models that more closely resemble the blast events occurring in the battlefield with the use of detonated explosives and/or open field settings when possible.

In this study, we aimed to identify the specific effects of repeated exposures to *explosive-driven blasts* utilizing a well characterized explosive driven blast device, the Blast Wave Generator (BWG)^19–22^ on neurological, behavioral, molecular, and possible neurohistologic outcomes suggestive of a higher risk for long-term neuropathological and clinical sequelae. Overall we wish to better understand the relative role of repeated blast exposure (e.g. repeated exposure to a blast overpressure wave as produced through the detonation of explosives) on the mammalian brain to provide a possible experimental basis for future research designed to improve detection/monitoring, treatment, and prevention of blast-related brain injury for Service Members and civilian populations at higher risk to be exposed to these catastrophic events.

## Results

### Blast characteristics

**Figure 1:**
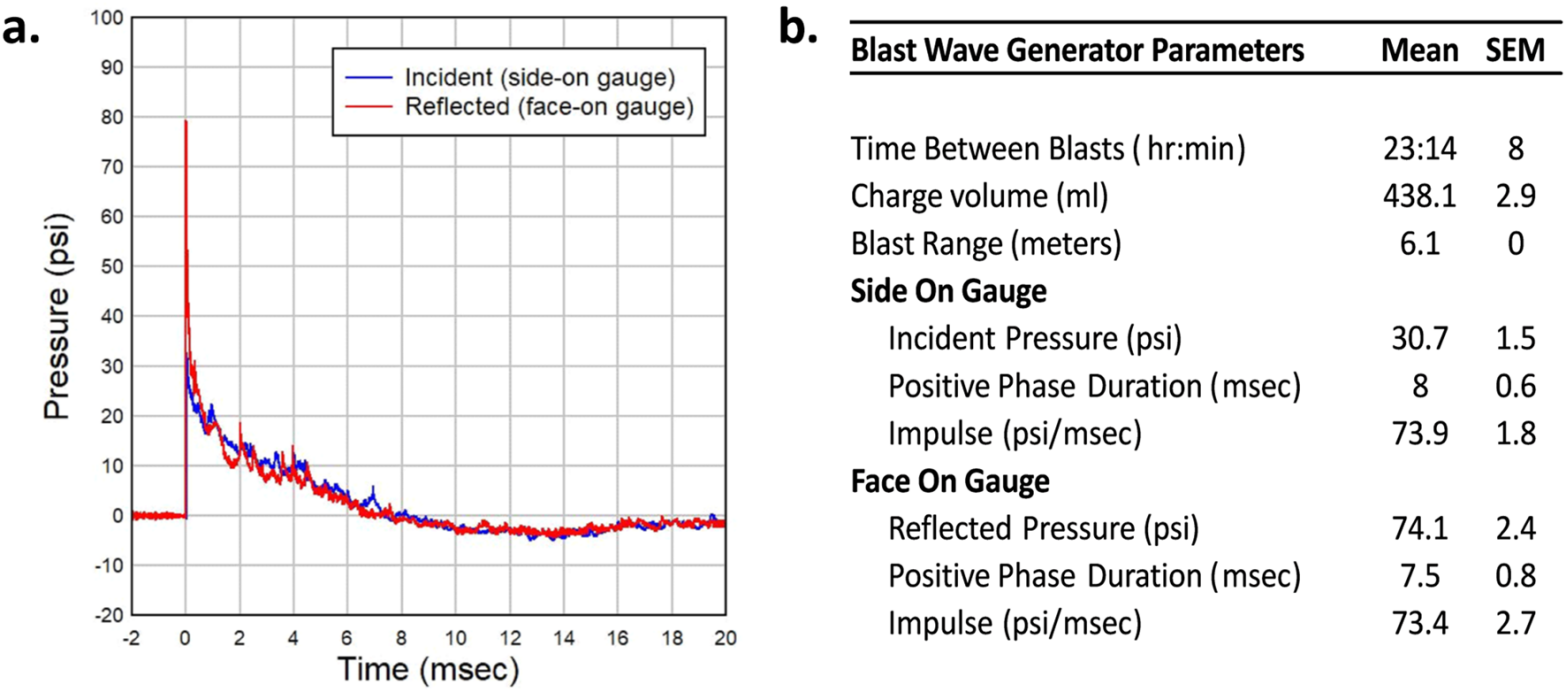
Blast Wave characteristics. (**a**) A representative pressure vs time waveform as produced by detonation of explosives within the Blast Wave Generator and measured with both side-on and face-on recording gauges. This waveform demonstrates a peak incident overpressure around 30 psi with a positive phase duration of approximately 8 ms. (**b**) Summary output data from Blast Wave Generator as recorded at time of blast.

Recordings confirmed an average peak incident pressure over all 24 blast events of 30.7 psi (SEM 1.5) with average positive phase duration of 8 ms (± 0.6) with an average time between blasts of 23 h 14 min (Fig. 1a,b).

### Effects of double blast exposure on behavioral outcomes

#### General health and neurological scores

**Figure 2:**
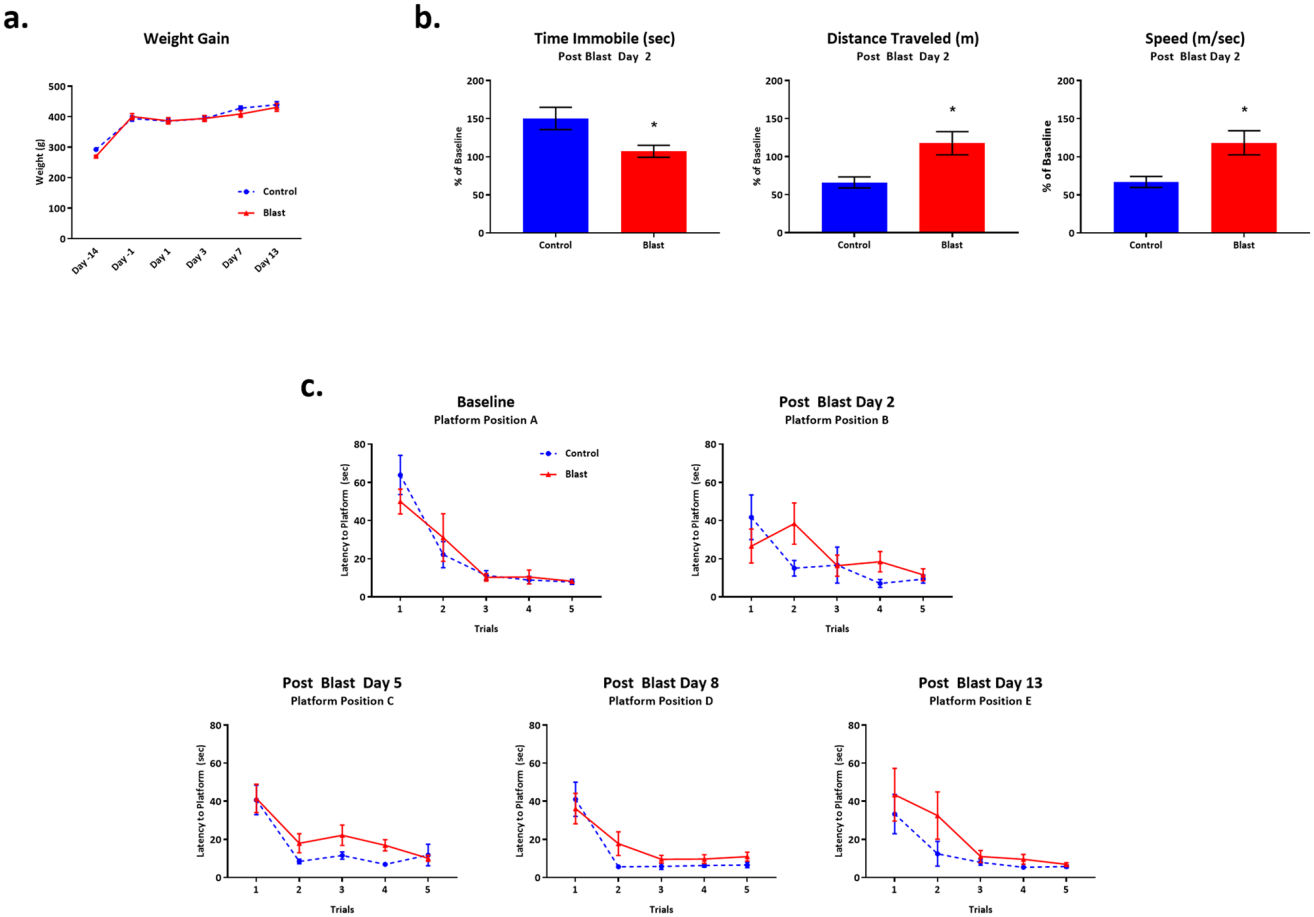
Behavioral outcomes following Double Blast Exposure. (**a**) No significant differences in weight gain were noted between 2 × B and Ctl over the course of the study, n = 12 per group. (**b**) Two days following the second exposure to blast, Open Field analysis revealed that blast exposed rats traveled significantly further, with greater speed, and spent less time immobile during the 5 min test session as compared with controls, n = 12 per group. * indicates *p* value < 0.05 as determined by 2-tailed, unpaired *t* test. Bars represent % of baseline for each group. Error bars represent standard error of the mean. (**c**) No significant differences in latency to find the hidden platform on the Morris water maze were noted between 2 × B and Ctl rats at all time points. n = 12 per group.

We did not observe any remarkable neurological differences between 2 × B vs. Ctl rats between Days 2–14 following double blast exposure. Both groups gained weight at the same pace for the course of the study (Fig. 2a). We observed no differences in basic neurological functions from baseline through Day 14. One blast exposed rat out of 12 demonstrated a loss of basic startle reflex (response to hand clap as part of Neurological Score assessment) at Day 1 post blast exposure, but it returned to normal by Day 3.

#### Open field

At Day 2 post-blast, we observed differences between 2 × B vs. Ctl rats in 3 measures of locomotor activity: distance travelled (*p* = 0.01), speed (*p* = 0.01), and time immobile (*p* = 0.02). Blast exposed rats traveled significantly further, with greater speed, and spent less time immobile during the 5-min test session as compared with controls (Fig. 2b). Essentially, these findings show that the 2 × B rats were more active than Ctl rats, which has been previously reported for rats exposed to multiple blasts^23^. Open Field analysis was only conducted at a single time point following blast or control related procedures to try to reduce habituation to the test environment^23^. It is interesting to note that it appears the Ctl rats may have habituated to the test environment as compared to the 2 × B rats since their performance decreased from baseline by nearly 50% in measures of distance travelled and average speed and their time spent immobile went up by nearly 50%. This further emphasizes the greater overall activity shown by the 2 × B rats.

#### Water maze

No significant differences were observed between 2 × B vs. Ctl rats at any time point in latency to find a hidden platform in the Morris Water Maze test of spatial memory as analyzed with 2-way repeated measures ANOVA. Rats were tested prior to blast exposure and again at 4 time points following blast and performed equally well at each test session (Fig. 2c).

#### Gait

We analyzed 13 separate gait parameters using the CatWalk XT 9.1 automated digital gait analysis system. Parameters examined included the following: stance (s), print length (cm), print width (cm), print area (cm^2^), print intensity (force), swing time (s), swing speed (cm/s), stride length (cm), base of support (cm), overall speed (cm/s), number of steps, cadence (steps/s) and regularity index (interlimb coordination). Each parameter was analyzed at each of 4 paws independently or in coordinated fashion depending on the measure. Rats were tested at baseline and again at 4 time points following blast exposure. With the exception of 2 individual data points from just 2 out of 13 parameters, there were no other significant differences between 2 × B vs. Ctl rats at each time point. The exception occurred on Day 3, in the parameters of swing speed in the right front paw (*p* = 0.03) and cadence (*p* = 0.03). By Day 6, there was no significant difference between 2 × B and Ctl on these 2 measurements. There were no differences in any other parameters at any other time points measured (Supplementary Fig. S1–S5 online).

### Molecular outcomes

#### *Effects of double blast exposure on pTau, all-Tau, related kinases and phosphatases in frontal cortex, hippocampus, cerebellum and brainstem* (Fig. 3)

**Figure 3:**
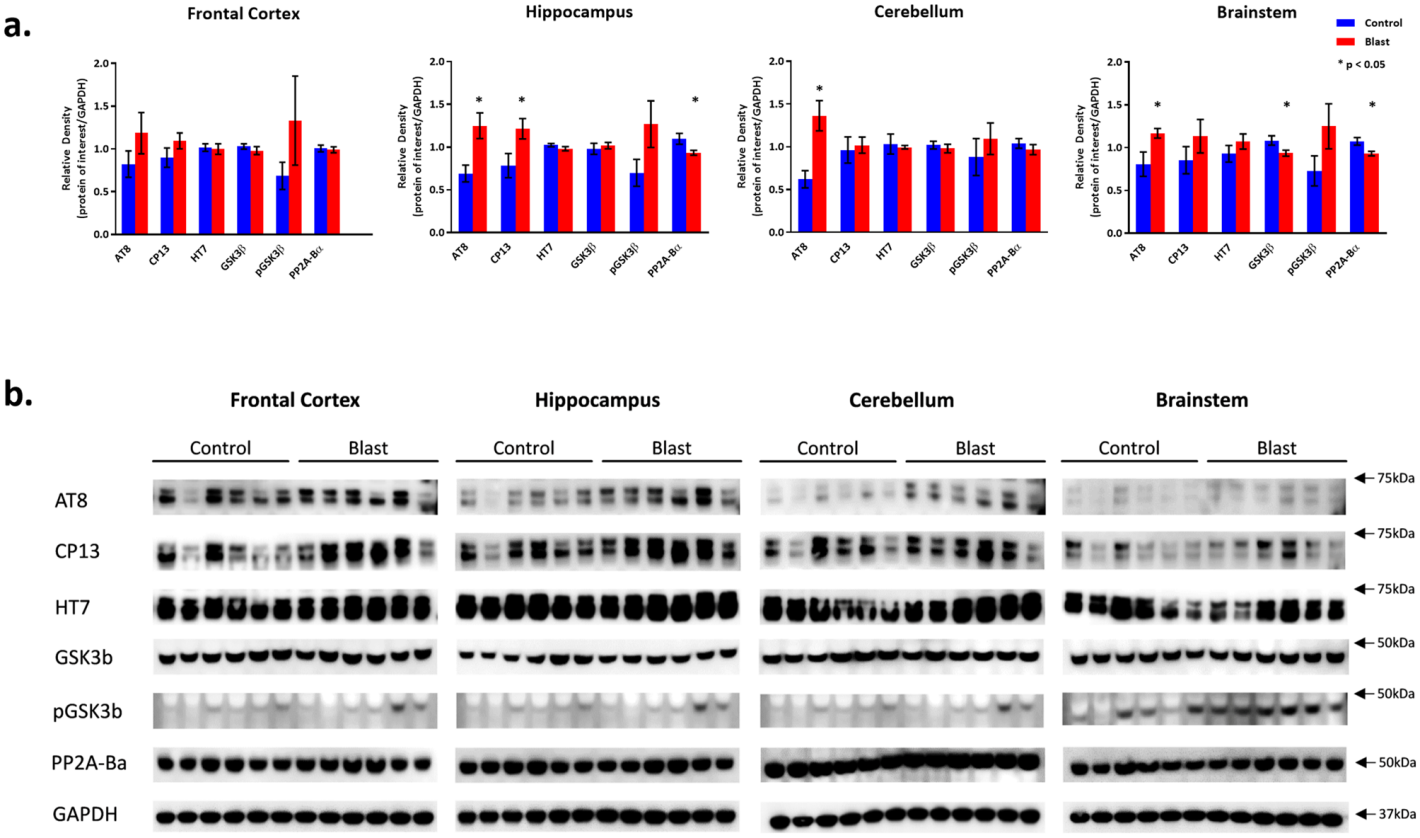
Tau and Related protein expression changes following Double Blast Exposure. (**a**) Histograms representing the densitometric ratio of levels of pTau (phosphorylated-Tau; AT8, CP13), total-Tau (HT7), kinases (GSK3β and pGSK3β), and phosphatase (PP2A-Bα) with respect to GAPDH as measured in the frontal cortex, hippocampus, cerebellum and brainstem in the brains of rats 15-days after an explosive-driven double blast (24 h apart) exposure, n = 6 per group, * indicates *p* values < 0.05 as determined by 2-tailed, unpaired, t-tests. Error bars represent standard error of the mean (SEM). (**b**) Representative western blots* for each antibody used. *For full length blots for each antibody, see Supplementary Fig. S10–S13 online.

Rats exposed to two 30-psi explosive blasts 24 h apart demonstrated increases in pTau measured with AT8 and CP-13 in all 4 brain regions investigated (though only significant in 3). AT8 levels were significantly higher in 2 × B vs. Ctl rats in the Hip (*p* = 0.01), Cere (*p* = 0.01) and BS (*p* = 0.04). AT8 level was also increased in the FCtx of 2 × B rats, though it did not reach significance. CP13 levels were significantly higher in 2 × B vs. Ctl rats in the Hip (*p* = 0.04). Increased levels of CP13, although not statistically significant, were also noted in the other regions. Total all-tau levels (HT7) were not significantly different between 2 × B vs. Ctl rats. Constitutively active GSK3*β* levels were not different between 2 × B vs. Ctl in the FCtx, Hip, and Cere; however, they were lower in 2 × B vs. Ctl in the BS (*p* = 0.04). More interestingly, there was an observable increase, though non-significant, in the level of pGSK3*β* (Ser 9) in all brain regions, indicating that the active phosphorylation of tau by GSK3*β* may have reached its peak already at the time point considered (15 days post-blast). In addition, we observed a significant decrease in PP2A-B*α* levels in 2 × B vs. Ctl in the Hip (*p* = 0.04) and BS (*p* = 0.02) and a slight, but non-significant, decrease in the Cere as well. Specifically, these results seem to be consistent with the increase of pGSK3*β* (Ser 9), which indicates a slowdown in active phosphorylation of tau at this specific time point and also as a possible switch to a more stable conformation change of tau.

#### *Effects of double blast exposure on injury associated protein expression* (Figs. 4, 5, 6)

**Figure 4:**
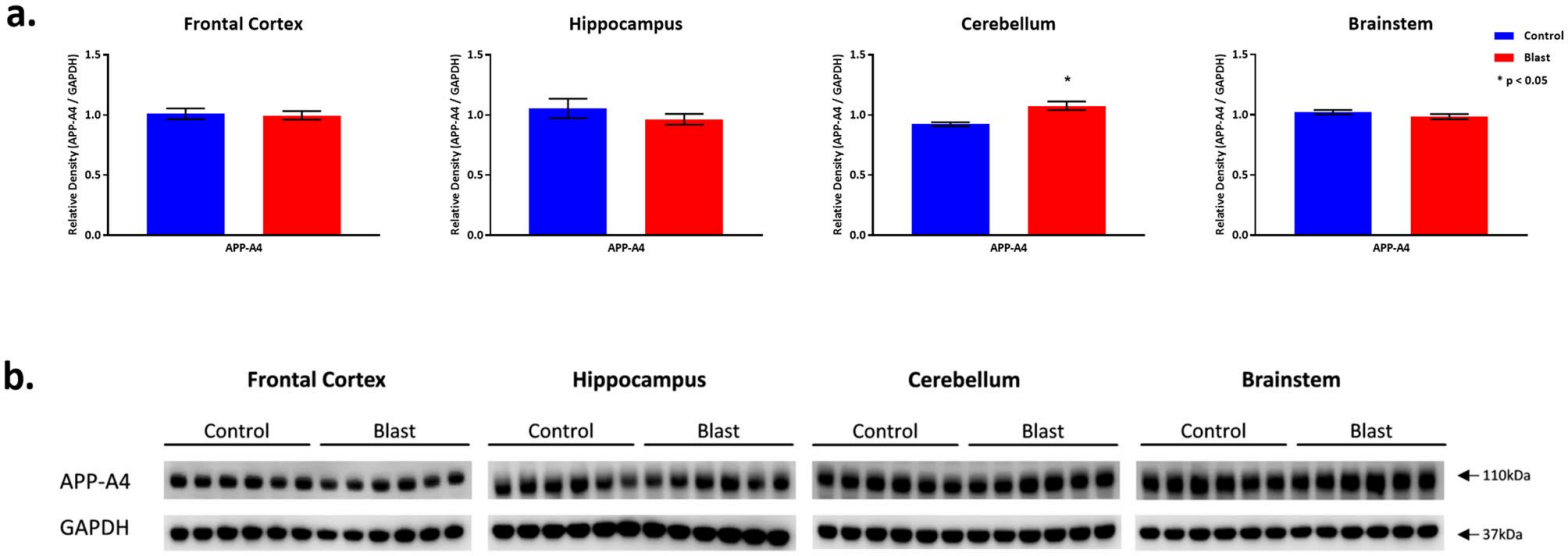
APP-A4 protein expression following Double Blast Exposure. (**a**) Histograms representing the densitometric ratio of levels of APP-A4 with respect to GAPDH as measured in the frontal cortex, hippocampus, cerebellum and brainstem in the brains of rats 15-days after an explosive-driven double blast (24 h apart) exposure, n = 6 per group. * indicates *p* values < 0.05 as determined by 2-tailed, unpaired, t-tests. Error bars represent standard error of the mean (SEM). (**b**) Representative western blots* for antibodies used. *For full length blots for each antibody, see Supplementary Fig. S10–S13 online.

**Figure 5:**
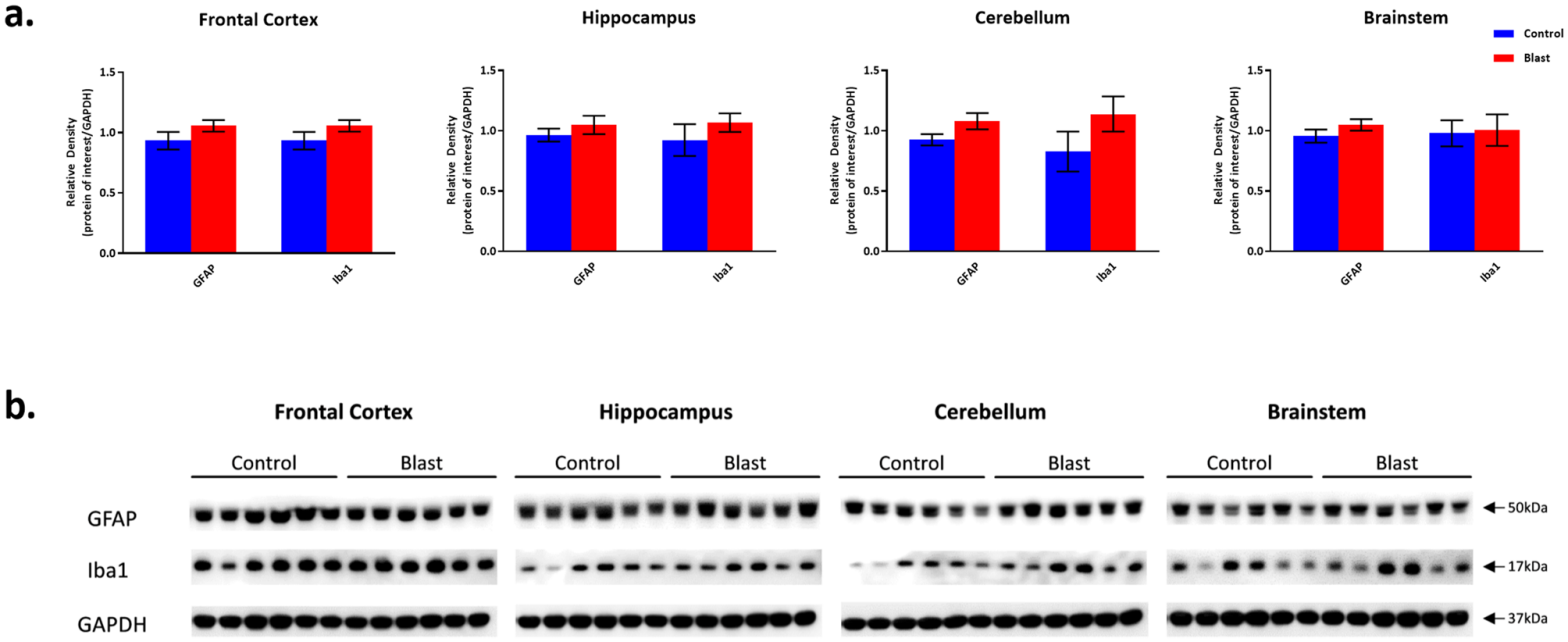
GFAP and Iba1 protein expression following Double Blast Exposure. (**a**) Histograms representing the densitometric ratio of levels of GFAP and Iba1 with respect to GAPDH as measured in the frontal cortex, hippocampus, cerebellum and brainstem in the brains of rats 15-days after an explosive-driven double blast (24 h apart) exposure, n = 6 per group. Error bars represent standard error of the mean (SEM). (**b**) Representative western blots* for each antibody used. *For full length blots for each antibody, see Supplementary Fig. S10–S13 online.

**Figure 6:**
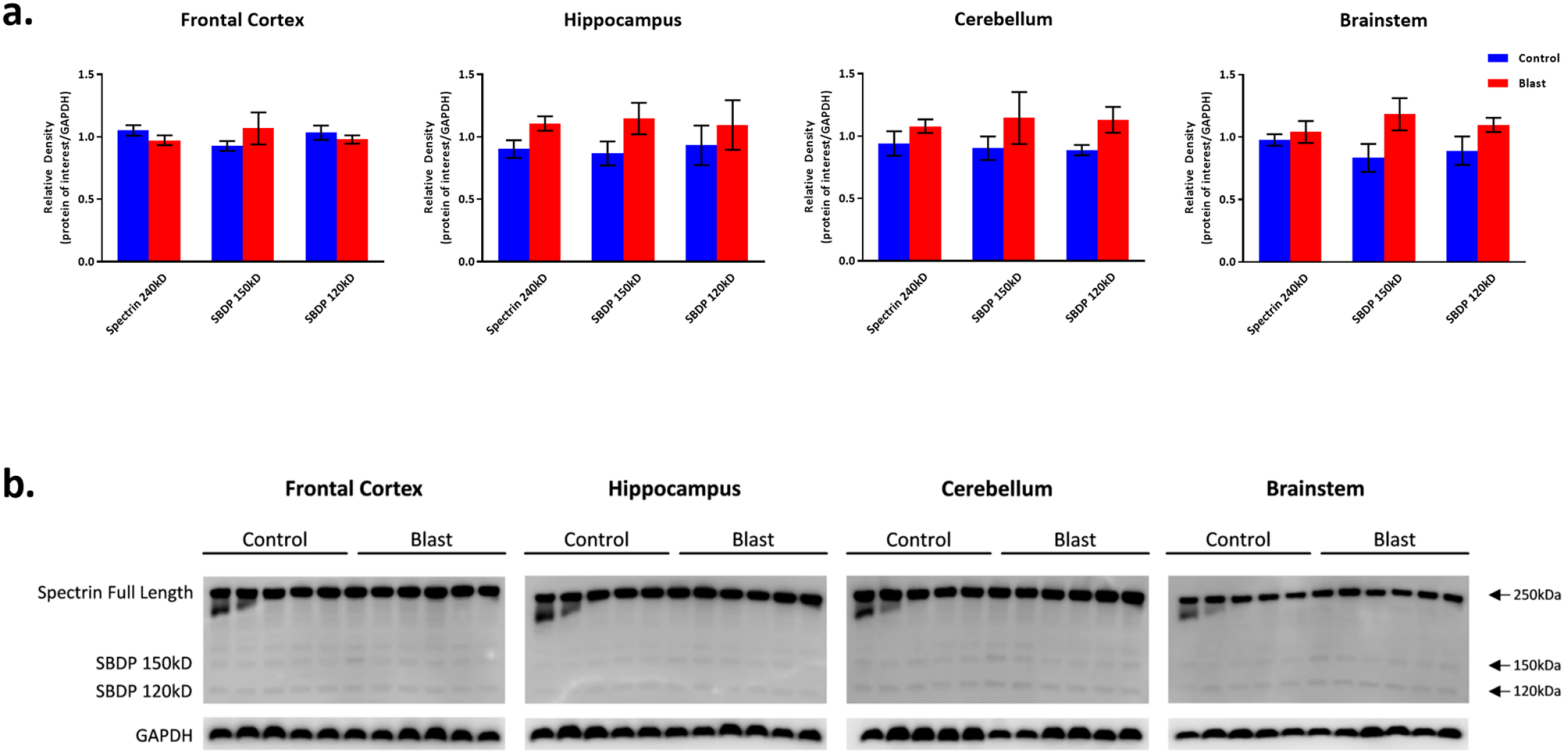
Spectrin and spectrin breakdown product expression following Double Blast Exposure. (**a**) Histograms representing the densitometric ratio of levels of full length spectrin (240kD) and spectrin breakdown products (SBDP 150kD and 120kD) with respect to GAPDH as measured in the frontal cortex, hippocampus, cerebellum and brainstem in the brains of rats 15-days after an explosive-driven double blast (24 h apart) exposure, n = 5* to 6 per group. Error bars represent standard error of the mean (SEM). *Only 5 control samples available in this experiment. (**b**) Representative western blots* for each antibody used. *For full length blots for each antibody, see Supplementary Fig. S10–S13 online.

APP expression level was unchanged in the FCtx, Hip, and BS between 2 × B and Ctl rats, but was significantly increased in the Cere (*p* = 0.0028) (Fig. 4). This is in line with other explosive^24^ and shock tube driven^25^ blast exposure studies in both mice and rats which report increased APP protein expression in various brain regions by western blot evaluation.

There were no significant differences between 2 × B and Ctl rats in GFAP, Iba1, αII-spectrin (full length protein 240kD) and both the 150kD and 120kD spectrin breakdown product levels across all examined brain regions (Figs. 5 and 6). There is a slight trend toward increase in the levels of GFAP and Iba1 in the blast group. These findings mirror those described by Baalman, et al.^26^. These authors demonstrated no differences from control in the same markers of injury in the cortex of rats 2 weeks following a *single* blast exposure.

### Neurohistological and Immunohistochemistry outcomes

Examination of H&E stained sections across the entire brain revealed no obvious morphological differences between 2 × B and Ctl. There was no evidence of hemorrhages or any other neuropathological signs of overt injury (Supplementary Fig. S6 online).

Immunohistochemistry performed by using AT8, CP13, APP, GFAP, and Iba1 did not show any type of microscopic brain lesion or abnormal protein accumulation (Supplementary Fig. S7—APP, and Fig. S8—AT8 online). This was not entirely surprising as many other groups have also reported no immunopositive staining for all of these selected proteins in either shock tube driven or explosive blast driven studies in mice, rats and swine^25–30^.

## Discussion

To the best of our knowledge, this experimental investigation for the first time analyzed delayed (after 2 weeks) behavioral, neurological, molecular and possible early neuropathological consequences of an *explosive-driven double blast exposure* rat model. The strength of using explosives as opposed to gas-generated blast simulators consists in the fact that this type of blast represents a much more comparable battlefield environment in which to analyze the *primary blast effects* on the body and brain in particular. This notion has been recently confirmed by Kawa, et al.^11^, who conducted a direct comparison between a gas-driven blast tube vs. explosive driven blast tube. In our experiment, using a more realistic explosive blast-exposure approach, we were able to provide novel data on the different levels of pTau in different regions of a mammalian brain (rat) and, at the same time, provide measurements of phosphorylation and de-phosphorylation protein levels in those same brain regions in support of the tau-related biochemical changes observed. Interestingly, our tau data roughly correlates with a recent in vivo PET study done in veterans exposed to blast in Iraq and Afghanistan demonstrating increased tau-tracer (T807/AV-1451) uptake in frontal, occipital, and especially cerebellar brain regions^31^.

Another intriguing finding of our study was the increased levels of APP in the Cere. While we did not observe any diffuse axonal injury (DAI) lesions, the fact that there was an APP increase in the Cere could represent a very specific marker of blast lesion since this cerebellar APP increase is rarely observed in other pathological conditions such as Alzheimer’s diseases (AD)^32,33^. In this case, a possible human study on Service Members exposed to blast using an APP tracer through PET-scanning could shed light on this specific aspect.

One of the most important findings of this study is the striking dissociation between the behavioral findings and elevated levels of pTau in Hip, Cere, BS (significant) and FCtx (trending) 2 weeks after explosive-driven double blast exposure. Previous studies have shown increased levels of pTau and some impairment in various behavioral outcomes in rodents after single or multiple gas or explosive driven blast exposures^34–37^. Each of these studies utilized lower pressures than 30 psi ranging from 6–21 psi, none used torso protection for the animals, the time post exposure ranged from 24 h to 30 days and they all reported behavioral changes along with increased pTau. Our findings, however, show increased levels of pTau 15-days post explosive-driven double 30 psi blast exposure while utilizing torso protection without any evident behavioral changes, at least for the functions/skills examined. We hypothesize that the neuropathological (molecular) changes initiated by increased levels of pTau in our specific model may not be clinically evident until a much later time point, that is, when a functional neuronal threshold has been crossed or additional neuropathological (histological) events accumulate (e.g. other blast events, a concussion, series of subconcussions). This “*delayed blast-effect*” hypothesis is in agreement with human epidemiological data showing that blast effects can indeed determine cognitive or psychiatric consequences much later after the initial exposure^4^. More specifically, we hypothesize that the possible persistence of higher pTau levels due to repetitive blast, along with other environmental or genetic factors could potentiate the initial blast-induced molecular changes and determine, cumulatively, long-term neuropathological effects. These blast-related delayed effects could then generate the manifestation of neuropsychiatric symptoms (e.g. anxiety or memory disorders, post-traumatic stress disorder, depression and suicidal ideation) only at a later moment in time, which is indeed a situation frequently observed in blast-exposed Service Members months or years after deployment. However, our hypothesis needs future confirmation by studying shorter and longer-term behavioral, molecular and neuropathological aspects detected at time points starting from a few hours until 12–18 months post-blast.

Moreover, these dissociated molecular-behavioral findings seem to suggest a very complex relationship between tau phosphorylation phenomena across different brain regions and other possible parallel molecular changes including brain repair and clearance mechanisms, neuroplasticity or physiological compensatory mechanisms. Discovering a one-to-one relationship between pTau increases and corresponding behavioral side effects may very much be a “*right place, right time*” situation. In support of that idea, our data suggest that the switch from activated kinases to deactivated kinases could be due to the fact that the phosphorylation of tau associated with GSK3*β* is transient and once a maximum level of phosphorylation is reached, it stops rather than moving to the hyper-phosphorylated states observed in other chronic neurodegenerative conditions. Similar results have been reported by Wang, et al.^38^ in rats following a single blast exposure where active GSK3*β* expression was stable and pGSK3*β* expression increased in the Hippocampus starting 1 day after blast and continuing out to only 6 weeks post-blast. This could be explained by the “buffering” or “clearance” activities of different cellular and subcellular systems activated in the brain as response to repeated traumatic events^39^. Furthermore, it would be relevant in the future to analyze other molecular aspects such as those associated with levels of phospho-*β*-catenin or phospho-Akt reported after gas-driven double blast exposure^40^.

One caveat of our study is that we chose a single time point for evaluation. Future studies investigating the tau phosphorylation status and related enzymatic activities at earlier and later time points following single and multiple blast exposures will be needed to determine if the tau phosphorylation observed in our model is a reversible phenomenon (or not) and which molecular mechanisms control it. Intriguingly, though, several studies in mice have demonstrated reversible tau phosphorylation at later time points following varied stressors^41–43^.

Forthcoming analyses should also incorporate measurements from peripheral blood biomarkers. These biomarkers could be used as signals of efficacy of specific pharmacological treatments that could delay, stop, or reverse the chronic long-lasting accumulation of pTau (if confirmed) and other possible pathological proteins in the brain related to exposure to blast. These blast-related tau phosphorylation phenomena, moreover, will also need to take into account a series of genetic and environmental factors such as sex, age, and pre-blast conditions that could contribute to further detrimental effects in addition to those triggered by the initial blast event on brain functions.

## Methods

### Experimental set-up

#### Animals

24 male Sprague–Dawley rats (Charles River Laboratories, Wilmington, MA, USA), 250–320 g (2–3 months of age) at arrival, were used. All rats were pair housed in the animal vivarium in standard rat cages in a 12 h light/dark reverse light cycle room with food and water provided ad libitum. Rats were acclimatized to the vivarium for 3 days prior to any handling. Rats were handled according to a protocol approved by the Institutional Animal Care and Use Committee (IACUC) at the Uniformed Services University (USU, Bethesda, MD) in compliance with the PHS Policy on Humane Care and Use of Laboratory Animals, the NIH Guide for the Care and Use of Laboratory Animals, and all applicable Federal regulations governing the protection of animals in research.

#### Study time course

**Figure 7:**
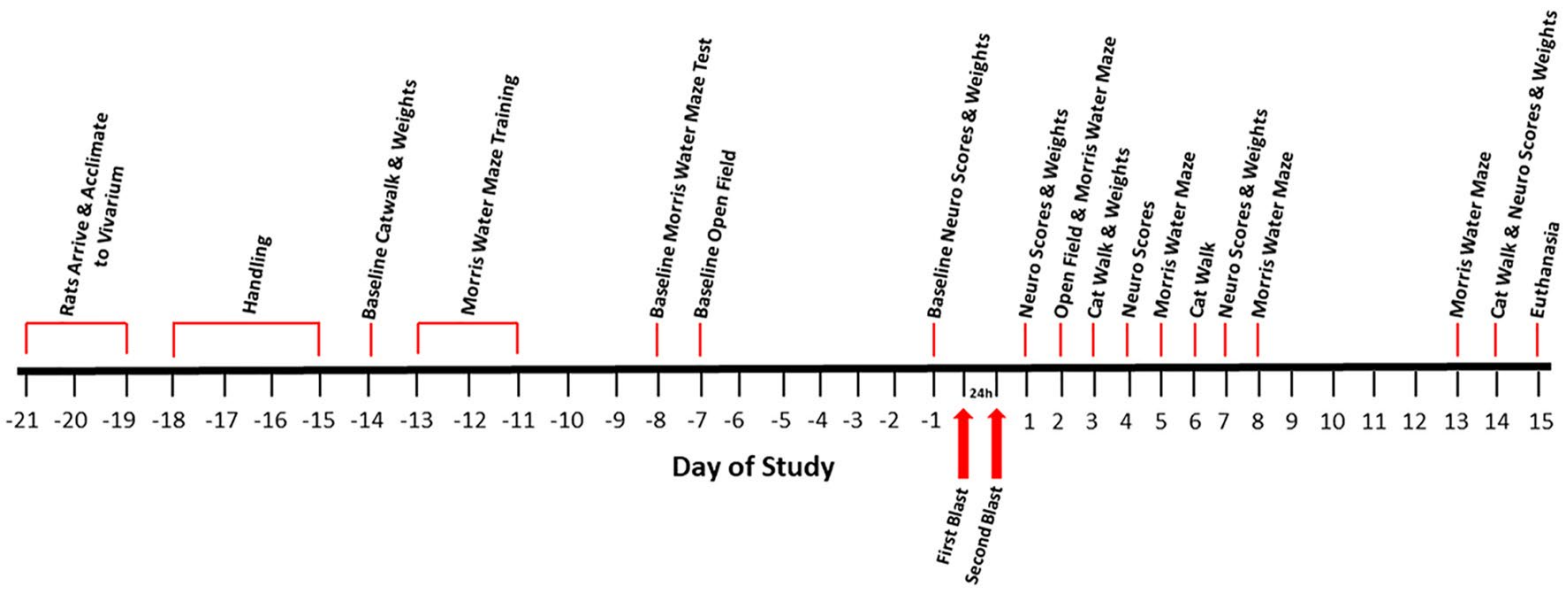
Experimental Timeline. A schematic diagram of the time course for this study. Time interval between blasts is 24 h.

Rats arrived approximately 21 days prior to the first blast day (Fig. 7). Following acclimation to the vivarium for 3 days, rats were handled for 4 days and baseline evaluations were conducted on all rats from Day − 14 to Day − 1 prior to the start of blast or control procedures. Rats were weighed and divided into 2 groups: explosive-driven double blast-exposed (2 × B) (rats exposed to two blast events, 24 h apart, *n* = 12) and sham control (Ctl) (rats exposed to an equivalent amount/quality of handling and anesthesia, but not to actual blast, *n* = 12). At Day − 14, rats began baseline behavioral tests for neurological functions (Neurological Severity Score, Neurobehavioral Scale, and an adapted Neurological Exam), cognitive function/spatial learning (Morris Water Maze), locomotor activity (Open Field), and gait analysis (Catwalk, Noldus Information Technology, Inc., Leesburg, VA, USA). Behavioral tests are described in detail below.

Baseline evaluations concluded on Day − 1. On Day 0, rats were transported to the blast site for the first of 2 blasts or blast-control exposures. Twenty-four hours later, rats were again transported to the blast site for the second exposure. From Day 1 to Day 14, rats were evaluated on the same behavioral tests used prior to the blast. On Day 15 all rats were euthanized. Blast exposures, related control procedures, and behavioral evaluations were carried out during the dark phase of a reversed 12-h light/dark cycle.

### Blast conditions

#### Blast site and transportation

Rats were transported back and forth on two consecutive days from the USU vivarium to an US Army range within relatively short driving distance. This is an adjunct research site approved for use by the IACUC of USU, Bethesda, MD. Transportation was provided in a climate controlled van dedicated for animal transport. Temperature and humidity was monitored throughout transportation. Food and water in the form of gelatinized Napa Nectar (SE Lab Group Inc., Napa, CA, USA) was provided to the rats while in transport. Water bottles were returned to each cage upon arrival at the test site and upon return to the vivarium. While at the test site, the rats were kept within a climate controlled building which was located at a safe distance from the blast with food and water access.

#### Blast set up

**Figure 8:**
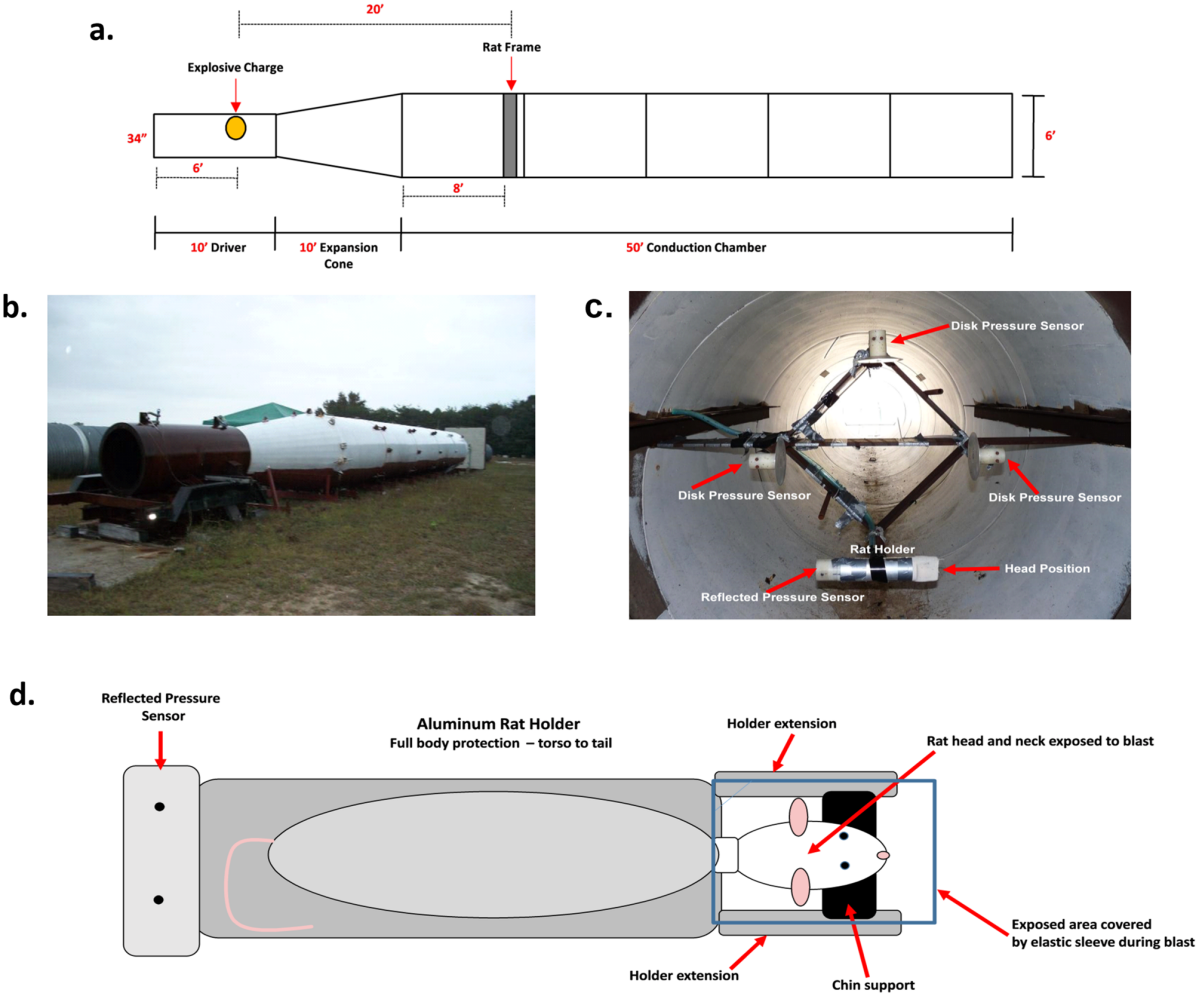
Blast tube parameters. (**a**) A diagrammatic scheme of the blast-tube used for this study. (**b**) The exterior aspect of the explosive-driven blast-tube used for this experiment. (**c**) A cross section of the inside of the BWG at the level of the rat holder and pressure gauges. This picture is taken from the perspective of the explosive charge located in the driver section of the BWG. In this picture, the rat holder is placed on the lower corner of the frame; disk pressure gauges are placed on the remaining three corners for the recording of incident (side-on) pressure. A “face-on” gauge, for the recording of reflected pressure, is fitted at the tail end of the rat holder (visible as a small spot at the left end of the holder in the picture). Rat holder and pressure gauges are equidistant from the explosive charge. During the actual blast exposures, rats were placed 2 at a time in the frame at the top and bottom vertices with the disk pressure gauges on the right and left vertices. (**d**) Schematic of the aluminum rat holder and rat position within the holder from the perspective of the explosive charge.

All blast exposures were carried out by contractors from ORA (ORA, Inc. Fredericksburg, VA). Blasts were generated within a 6′ diameter, 70′ long explosive-driven blast wave generator (BWG). This generator has been validated with a large animal (Yorkshire swine) model resulting in blast-induced injury at both the whole body and brain levels^19–22^. The BWG is comprised of 3 sections (Fig. 8a,b):

- the driver section, about 3′ wide and 10′ long where the explosive charge is placed;
- a 10′ long expansion cone;
- a conduction chamber, 6′ wide and 50′ long where the target is placed.

The test rats were positioned off-axis from the explosive within cylindrical aluminum holders designed to minimize primary blast-related lung injury and tertiary (acceleration) blast mechanisms. In these holders, only the rat’s head and neck were exposed to the direct impact of blast’s shock waves. The head rested on a chin support fitted between two extensions from the holder. The exposed end of the holder was covered by a stretchable elastic sleeve to protect the head from superficial singeing by the blast’s rapid heat flash. The cylindrical aluminum holder was firmly attached to a diamond shaped holding fixture (Fig. 8C) within the shock tube, with the back of the rat and top of the skull facing the charge (Fig. 8D). The use of the BWG and holding fixture arrangement allowed for the use of significantly smaller explosive charges than would be required in an open air test event. Additionally, the experimental setup allowed for the exposure of the rats to a “pure” blast event without reflected shock fronts from the ground or other surfaces. The use of this setup also allowed for the isolation of the primary mechanism of injury by blast with placement of the rat outside of the fireball to prevent injury due to fragments or increased impulse generated by the higher density of the fireball. Pressure transducers were placed in close proximity to the rat and registered pressure as a function of time following blast, 2 side-on disk gauges to record incident pressure and 1 face-on gauge to record reflected pressure were used. The animals and pressure gauges were equidistant from the center of the cross section of the tube and from the source of the blast. The pressure–time recordings were used to confirm the *impulse* generated by each blast (impulse being defined as the overpressure-time integral in the time interval between the initial pressure rise and the first return to ambient pressure)^44^.

#### Blast exposure

Prior to blast exposure, twelve (12), rats were deeply anesthetized by *i.p.* administration of a mixture of ketamine (80 mg/kg) and xylazine (10 mg/kg) and placed into the cylindrical aluminum holder to minimize the blast impact on other organ systems. The holder was attached to the fixture within the conduction chamber as described above. Rats were exposed 2 at a time to a shock wave of about 30-psi (~ 207 kPa) incident overpressure and 8–10 ms positive-phase duration, as generated by the detonation of high explosive (sensitized liquid nitromethane) within the blast wave generator. The explosive charge was placed within the driver section of the tube at a 20′ distance from the fixed rat holder. Preliminary blasts (calibration shots) were carried out with pressure transducers, but without test rats, to establish the charges needed to obtain the target peak pressure of 30-psi (~ 207 kPa). We chose this pressure based on our pilot studies examining the dose response (20–50 psi or 138–345 kPa) and lethality limits for single blast exposure in rats. These studies revealed that with torso protection, pressures around 30-psi (~ 207 kPa) provided the least amount of lung injury and greatest survival (unpublished pilot data, see Supplementary Fig. S9 online for lung injury comparison) and the lethality observed in torso protected rats exposed to a single blast between 25–35psi was 6% as compared with a lethality of 29% in torso protected rats exposed to a single blast between 42–50psi. In addition, it has been determined that 30-psi (~ 207 kPa) is also the threshold for lung injury in humans^45^. In the present study, each rat was exposed twice to a 30-psi blast wave, 24 h apart. We chose a time interval of 24 h since we had evidence from our pilot studies that rats exposed to a single 30-psi exposure have normal appearance and neurological behavior 24 h after the exposure (with the exception of acoustic deficit as tested with acoustic startle response, which normally recovers over the course of 4–6 weeks). Consequently, we decided that an interval of 24 h from the first exposure was the minimal acceptable time to avoid lethality from the second exposure.

Additionally, throughout our single exposure pilot studies at 30psi, no significant behavioral deficits (neurological scores, open field, Morris water maze, rotarod, novel object recognition, Barnes maze) were observed from 24 h to 28 days post exposure. We did observe changes in Acoustic Startle Response in the acute recovery phase which gradually returned to normal and a few gait related parameters which were also transient in nature, returning to normal after 2 weeks (pilot studies, data unpublished).

#### Control-blast procedures

Twelve rats were handled in the same way as the blast exposed rats, including transportation and administration of anesthesia, but they were not exposed to blast.

#### Post-blast procedures

Following exposure to blast, rats were removed from the holders, brought back to the protected and climate controlled building, checked for immediate evidence of injury (bleeding from eyes, ears or nose, respiratory distress, burned/singed fur or whiskers), and allowed to recover from anesthesia in their home cages. Rats were continuously monitored for at least 2 h immediately after blast exposure or blast-control procedures. Once rats had recovered from anesthesia, they were also checked for neurological status (gait, reflexes, and signs of paralysis). All rats were brought back to USU, Bethesda, MD within 12 h of their departure. Rats were again monitored for pain and distress at the return to the vivarium, twice the following day and daily thereafter for the duration of the study. Rats were evaluated using the pain/distress scoring guidelines, based on a previously published scoring system^46^. Evaluation included changes in appearance, respiration, provoked and unprovoked behavior. Body weight was measured 1, 3, 7 and 14 days following exposure to blast.

### Behavioral evaluations

#### Neurological functions

All rats were evaluated for basic neurological function at baseline during the 2 weeks prior to blast and again at Day 1, Day 4, Day 7 and Day 14 post-blast or associated control procedures. The following neurological scales were used on all animals in the study: Neurological Severity Score, NSS^47^, Neurobehavioral Scale, NBS^48^, and an adapted Neurological Exam^49^. The three neurological damage scales assess a range of basic reflexes including righting, startle, balance as well as general movement and muscle tone.

The NSS is scored either 1 (unable) or 0 (able) and examines the ability of the rat to perform in the following tasks. 1) Exit from a circle 50 cm in diameter. 2) Righting reflex. 3) Hemiplegia. The rat is pushed back and forth at the shoulders and should resist equally in both directions. A point is given if resistance is not equal. 4) Hind limb flexion. A normal rat when raised by the tail will extend both hind limbs, reaching upwards. If the rat flexes a hind limb, a point is given. 5) Walk in a straight line and ability to move. A point is allotted for each function. 6) Startle reflex to a loud noise about 20 cm above the rat’s head. The rat should flinch heavily. If it does not, a point is given. 7) Pinna reflex to touching the external auditory meatus with a cotton tipped ear swab. If the rat does not shake its head back and forth, a point is assigned. 8) Seeking behavior and ability to stand. If a rat has lost its seeking behavior (a normal rat will walk around and sniff unknown objects), the rat receives a point. If the rat is prostrated, another point is given. 9) Placing reflexes. The rat is lifted 5 cm off the ground by the tail and back. The animal should “reach” for the ground and place its limbs on the floor with palms facing the ground. A point is allotted for each limb’s inability to place. 10) Balance beam and beam walking. The rat is placed on a 1.5 cm wide beam. A point is given if the rat falls off within 60 s, another point if that fall was within 40 s, and another point if that fall was within 20 s. The rat is then placed on beams 2.5 cm, 5.0 cm, or 8.0 cm in width. A point is given for failure on any width.

The NBS is divided into four categories. Rats are graded on a scale of 0–4, with 4 being normal and 0 being non-functional. The categories are: (1) Forelimb flexion upon suspension by tail. (2) Decrease resistance to lateral pulsion. (3) Circling behavior upon spontaneous ambulation and (4) Ability to stand on an inclined plane.

The final scale is very simplified neurological exam. A grade of 0 is given to a normal rat. A grade of 1 is given to a lethargic rat. A grade of 2 is given to a rat with clear signs of paresis, but with the ability to walk. A grade of 3 is given to a rat with the inability to walk. A grade of 4 is given to a dead rat.

#### Locomotor activity

A general evaluation for locomotor activity was measured using the Open Field Test. Rats were tested at baseline during the 2 weeks prior to blast and again at Day 2 after the last blast exposure or associated control procedure. Rats were placed in a circular arena (74 cm diameter × 16.5 cm wall height) with bedding and allowed to explore for 5 min. Tests were conducted in ambient lighting and the bedding was mixed between animals to evenly distribute any smells carried over from the previous runs. Activity while in the arena was videotaped and then analyzed using the Any-Maze video tracking system (Stoelting Co., Wood Dale, IL, USA). Time spent immobile, total distance travelled and speed were assessed. Open Field analysis was only conducted at a single time point following blast or control related procedures to try to reduce habituation to the test environment^23^. Data was expressed as percent of baseline on Day 2 post blast, n = 12 per group.

#### Spatial learning

Cognitive function as measured by spatial learning ability was evaluated using the Morris Water Maze (MWM). Rats were trained and tested at baseline during the 2 weeks prior to blast and again at Day 2, 5, 8, and 13 post-blast or associated control procedures. The maze consisted of a water tight pool approximately 135 cm in diameter and 60 cm deep. The pool was equipped with a built in heater and thermostat to control the water temperature. The water temperature was kept at approximately 22–24 °C. It was filled with water to a depth of approximately 50 cm. A circular platform approximately 12.5 cm in diameter was placed 1.5 cm below the surface of the water. The water was made opaque by adding 250 ml of white non-toxic paint to the pool (Washable Poster Paint, Palmer Paint Products Inc., Troy, MI, USA). Large shapes were placed on each wall around the room to provide visual cues to the rats to help find the platform. The platform was placed in one of four quadrants of the pool, not in the center. The test was conducted in a dimly lit room to reduce reflections on the water surface. The test was videotaped and evaluated using the Any-Maze video tracking system (Stoelting Co., Wood Dale, IL, USA).

Each training or test day consisted of 5 trials conducted as follows: the rat was released into the pool with the nose facing the wall of the pool from 1 of 5 directional locations around the pool (N, S, E, W, and either NE, NW, SE or SW) chosen at random. The rat was allowed to swim up to 120 s in order to find the hidden platform. If the rat failed to find the platform in that time, it was gently guided to the platform. Once on the platform it was allowed to remain there for 30 s. The rat was then picked up gently and quickly dried with a towel and placed in a cage lined with a water-circulating heat pad set to 25 °C and dry towels. The rat remained in the warmed cage for 60 s before the start of the next trial. At the completion of the trials for the day, each rat was thoroughly dried and placed in the warmed cage for several minutes before being placed back into its own home cage. The platform position remained the same during the training sessions, but was changed at the beginning of the baseline and each post-blast test session. Position of the platform was changed to evaluate how well the rats learned the task to find the hidden platform and then within each day how quickly they memorized the new location. Latency to find the platform was recorded for each trial and a daily average latency was analyzed for each rat.

#### Gait

Gait analysis was carried out using the CatWalk XT 9.1 automated digital gait analysis system (Noldus Information Technology, Inc., Leesburg, VA, USA). Rats were tested at baseline during the 2 weeks prior to blast and again on 3, 6 and 14 days after exposure to blast or associated control procedures. The apparatus consists of a 1.3-m-long glass plate illuminated from below with dim fluorescent light and from above with red light. The walkway is enclosed on either side by solid black walls. In a darkened room, rats are encouraged to voluntarily walk along the platform. As the paw contacts the glass, the light is reflected producing illuminated footprints which are captured by a high speed video camera from below the glass surface. The software visualizes the prints and calculates statistics related to print dimensions and the time and distance relationships between footfalls^50,51^. Rats were first acclimated to the darkened room for 15 min and then individually placed on the walkway. They were allowed to walk freely back and forth across the platform and had to complete a minimum of 3 passes of the walkway at each test day. Footprints could be captured in either direction. Walk speed variation of 60% as recorded by the software was the cutoff level for an acceptable pass of the walkway. The apparatus was cleaned between each animal. Analysis of foot prints, as recorded during the test, were used to assess several endpoints, including: print length, width and area; stance phase (time paw is in contact with glass surface), mean intensity (pressure of paw on the surface); swing time and swing speed (time paw is not in contact with glass surface and the speed between 2 consecutive placements of the same paw), stride length (distance between 2 consecutive paw placements of the same paw); front and hind base of support (distance between front or hind limbs respectively); cadence (frequency of steps); regularity index (interlimb coordination); average speed and number of steps^52^.

### Tissue collection

On Day 15, rats were deeply anesthetized by *i.p.* administration of a mixture of ketamine (80 mg/kg) and xylazine (10 mg/kg). Upon absence of muscle reflex from a toe pinch, rats were euthanized via thoracotomy and half of the rats from each group (2 × B, *n* = 6 and Ctl, *n* = 6) were perfused transcardially with 0.9% saline for 5 min, brains removed and flash frozen in chilled isopentane and stored at  − 80 °C for protein analysis. The other half of rats (2 × B, *n* = 6 and Ctl, *n* = 6) were transcardially perfused with 0.9% saline followed by cold fixative solution made up of 0.1 M phosphate buffer, pH 7.4 and 4% paraformaldehyde for 5 min. Brains were removed and post-fixed in the same fixative overnight at 4 °C. The next day brains were transferred to 20% sucrose for cryoprotection and kept at 4 °C until they equilibrated with the solution (sunk to bottom). Brains were then flash frozen in chilled isopentane, and stored at − 80 °C for future use.

### Protein extraction and western blot (WB) procedures

Fresh frozen brains were cut at 100 µm thick sections on a cryostat and 4 brain regions, Frontal Cortex (FCtx), Hippocampus (Hip), Cerebellum (Cere), and Brainstem (BS) were carefully microdissected from these sections^53^. Dissecting the brain in different regions allowed us the opportunity to possibly observe different levels of vulnerability across different anatomical regions of a mammalian brain. Approximately 100 mg of tissue was collected from each region. Samples were homogenized in glass dounce homogenizers with ice cold lysis buffer (1 ml/100 mg tissue) containing the following: 50 mM Tris–HCl (pH 8), 1% Igepal, 150 mM NaCl, 1 mM EDTA, 1 mM PMSF, 1 mM NaF, 1:100 protease inhibitor cocktail (Sigma-Aldrich, P2714, St. Louis, MO, USA). Samples were centrifuged at 12,000 g for 20 min and supernatants collected, aliquoted and frozen at − 80 °C. Total protein content from each brain region was determined using the Micro BCA assay (Thermo-Fisher Scientific, 23235, Waltham, MA, USA). 20 µg of protein per sample, for all brain regions listed, were loaded on Novex Nupage 4–12% Bis–Tris Gels (Life Technologies, NP0329, Carlsbad, CA, USA) and were electrophoresed at 200 V constant for 30 min. For high molecular weight proteins (αII-Spectrin, 240 kD and SBDP, 120 and 150 kD), 20 μg of protein per sample was loaded on Novex Nupage 3–8% Tris Acetate Gels (Life Technologies, EA03785, Carlsbad, CA, USA) and were electrophoresed at 150 V constant for 60 min. Gels were transferred to PVDF membranes using the iBlot2 dry transfer method (Life Technologies, IB21001, Carlsbad, CA, USA). Membranes were blocked in 5% milk in 1 × TBST for 1 h at room temperature. Primary antibodies (see Primary antibodies section below) were diluted to the appropriate working concentrations in 5% milk in 1 × TBST or in 1 × TBST alone (for AT8 only) and incubated on the membranes overnight at 4 °C. Membranes were then rinsed 3 × 5 min in TBST. Appropriate HRP tagged secondary antibodies (see Secondary antibodies section below) were diluted 1:2000 in 5% milk in 1 × TBST or in 1 × TBST alone (for AT8 only), and incubated on the membranes for 1 h at RT. Membranes were rinsed 3 × 5 min in TBST and 1 × 5 min in TBS. Membranes were incubated with chemiluminescent substrate (SuperSignal West Pico Chemiluminescent Substrate, Thermo-Fisher Scientific, 34577, Waltham, MA, USA) for 1 min and imaged on the LiCor C-Digit Blot Scanner (LiCor Biosciences, Lincoln, NE, USA). All membranes were stripped one time with Restore Plus Stripping Buffer (Thermo-Fisher Scientific, 46430, Waltham, MA, USA), for 10 min, rinsed with TBS and processed for immunoblotting as described above using GAPDH (1:40,000, Millipore-Sigma, AB2302, Billerica, MA, USA) for the loading control. Densitometry was performed with NIH ImageJ software (2.0.0) with all protein signal intensities normalized to GAPDH signal intensity.

#### Primary antibodies

To examine possible abnormal levels of soluble phosphorylated-tau (pTau) at different epitopes, the levels of AT8, which recognizes tau phosphorylated at S-202 and T-205 (1:500, Thermo-Fisher Scientific, MN1020, Waltham, MA, USA) and CP13, which recognizes tau phosphorylated at S-202, (1:250, Gift of Peter Davies’ Lab, Albert Einstein College of Medicine, Bronx, NY, USA), were measured. In addition, to examine levels of soluble total-Tau, the level of HT7, which recognizes the full length isoform ~ 79kD (1:500, Thermo-Fisher Scientific, MN1000, Waltham, MA, USA), was measured. Moreover, we explored expression levels of kinase and phosphatase enzymes associated with the phosphorylation state of tau by measuring GSK3*β*, a constitutively active kinase responsible for phosphorylation of tau (1:500, Santa Cruz Biotechnology, sc-53931, Dallas, TX, USA)^54,55^, pGSK3β (Ser9), the deactivated kinase (1:500, Santa Cruz Biotechnology, sc-373800, Dallas, TX, USA)^56,57^, and PP2A-Bα, a primary tau phosphatase (1:500, Millipore-Sigma, 05–592, Billerica, MA, USA)^58,59^.

In addition, we measured levels of proteins known to be associated with neuronal injury and neuroinflammatory response: glial fibrillary acidic protein (GFAP) (1:1000, Leica Biosystems, NCL-L-GFAP-GA5, Newcastle Upon Tyne, UK), a marker for astrocytes and related neuroinflammatory phenomena^60^, ionized calcium-binding adapter molecule 1 (Iba1) (1:1000, Abcam, ab178847, Cambridge, MA, USA), a marker for microglial cells and their related activation^61^, and αII-Spectrin and its associated caspase and calpain mediated spectrin breakdown products (150kD and 120kD respectively) (1:40,000, Millipore-Sigma, MAB1622, Billerica, MA, USA) for their association observed in some TBI models^62–66^. Lastly, we measured levels of amyloid precursor protein (APP) (1:1000, Millipore-Sigma, MAB348, Billerica, MA, USA), a marker associated with diffuse axonal injury (DAI)^25,67–73^.

#### Secondary antibodies

The following HRP tagged secondary antibodies were used: Goat anti-mouse (1:2000, Abcam, ab97040, Cambridge, MA, USA), Goat anti-rabbit (1:2000, Abcam, ab97080, Cambridge, MA, USA), Goat anti-rat (1:2000, Thermo-Fisher Scientific, 62–9520, Waltham, MA, USA), and Rabbit anti-chicken (1:5000, Millipore-Sigma, AP162P, Billerica, MA, USA).

### Neurohistologic and immunohistochemistry procedures

Whole fixed frozen brains were divided into 5 blocks from frontal to caudal and each block serially sectioned in the coronal plane at 20 μm on a cryostat. Sections were mounted directly onto superfrost plus slides (Fisher Scientific, 12–550-15, Pittsburgh, PA, USA), 2 sections per slide.

Standard protocols were followed for staining with hematoxylin and eosin (HE) (Thermo Scientific, 6765001 and 6766009, Waltham, MA, USA) to identify possible gross morphological or neuropathological changes. Immunohistochemical analyses were performed using AT8, (1:250, Thermo-Fisher Scientific, MN1020, Waltham, MA, USA), CP-13, (1:100, Gift of Peter Davies’ Lab, Albert Einstein College of Medicine, Bronx, NY, USA), APP (1:200, Millipore-Sigma, MAB348, Billerica, MA, USA), GFAP (1:250, Leica Biosystems, NCL-L-GFAP-GA5, Newcastle Upon Tyne, UK Carlsbad, CA, USA) and Iba1 (1:100, Wako, 019-19741, Richmond, VA, USA). Slides were rinsed 3 × 5 min in 1 × PBS then incubated in endogenous peroxidase blocking solution (1 × PBS + 0.3% H_2_O_2_ + 0.001% Methanol) for 15 min at RT. Slides were rinsed 3 × 10 min in 1 × PBS then incubated in blocking solution (1 × PBS + 4% normal horse serum + 0.1% Triton X-100), for 1 h at room temperature. Primary antibodies were diluted to appropriate concentrations in 1 × PBS + 3% normal horse serum and incubated on the slides overnight at 4 °C. Slides were rinsed 3 × 10 min in 1 × PBS then incubated with appropriate biotinylated secondary antibody solution (R.T.U. Biotinylated horse-anti-rabbit or horse-anti-mouse IgG, Vector Laboratories, BP-1100 and BP-2000), Burlingame, CA, USA) for 1 h at RT. Slides were rinsed 3 × 10 min in 1 × PBS, then incubated with Vectastain Elite ABC Reagent followed by DAB chromogen according to manufacturers’ instructions (Vector Laboratories, PK-6100 and SK-4100, Burlingame, CA, USA) to visualize immunolabeling. Slides were counterstained with hematoxylin, dehydrated through a series of alcohols (70%, 95%, 100% 2 × 2 min each) followed by xylene (2 × 5 min), coverslipped with Permount (Fisher Chemical, SP-15, Fair Lawn, NJ, USA) and allowed to dry. Slides were examined on an Axio Imager microscope (Carl Zeiss Microscopy, Thornwood, NY, USA) and scanned on an Aperio AT2 slide scanner (Leica Biosystems, Buffalo Grove, IL, USA).

### Statistics

Data from WB densitometry and Open Field were analyzed by 2-tailed, unpaired *t*-tests. Data from Morris Water Maze and CatWalk were analyzed by 2-Way Repeated Measures ANOVA with Sidak’s Multiple Comparisons *post-hoc* test. Differences with *p* value < 0.05 were considered significant in all cases. Statistical tests were performed using GraphPad Prism version 7.03 for Windows (GraphPad Software, La Jolla, CA).

### Ethics approval

All animal work was approved by the Institutional Animal Care and Use Committee at the Uniformed Services University (USU, Bethesda, MD, USA) in compliance with the PHS Policy on Humane Care and Use of Laboratory Animals, the NIH Guide for the Care and Use of Laboratory Animals, and all applicable Federal regulations governing the protection of animals in research.

## Supplementary information

Supplementary Figure 1.

Supplementary Figure 2.

Supplementary Figure 3.

Supplementary Figure 4.

Supplementary Figure 5.

Supplementary Figure 6.

Supplementary Figure 7.

Supplementary Figure 8.

Supplementary Figure 9.

Supplementary Figure 10.

Supplementary Figure 11.

Supplementary Figure 12.

Supplementary Figure 13.

Supplementary Legends.

## Data Availability

The datasets used and/or analyzed during the current study and supporting the conclusions of this article are included in this article and in all supplementary materials provided. These datasets are also available from the corresponding author on reasonable request.

## References

[1] Elder G, Mitsis E, Ahlers S, Cristian A (2010). Blast-induced mild traumatic brain injury. Psychiatr. Clin. North Am..

[2] Hoge C (2008). Mild traumatic brain injury in U.S. soldiers returning from Iraq. N. Engl. J. Med..

[3] Wallace D (2009). Improvised explosive devices and traumatic brain injury: the military experience in Iraq and Afghanistan. Australas. Psychiatry..

[4] Rosenfeld J (2013). Blast-related traumatic brain injury. Lancet Neurol..

[5] Abbotts R, Harrison S, Cooper G (2007). Primary blast injuries to the eye: a review of the evidence. J. R. Army Med. Corps..

[6] Cernak I, Noble-Haeusslein L (2010). Traumatic brain injury: an overview of pathobiology with emphasis on military populations. J. Cereb. Blood Flow Metab..

[7] Cernak I (2017). Understanding blast-induced neurotrauma: how far have we come?. Concussion..

[8] Wolf S, Bebarta V, Bonnett C, Pons P, Cantrill S (2009). Blast injuries. Lancet.

[9] Agoston D (2017). Modeling the long-term consequences of repeated blast-induced mild traumatic brain injuries. J. Neurotrauma.

[10] Song H (2018). Linking blast physics to biological outcomes in mild traumatic brain injury: narrative review and preliminary report of an open field blast model. Behav. Brain Res..

[11] Kawa L (2018). A comparative study of two blast-induced traumatic brain injury models: Changes in monoamine and galanin systems following single and repeated exposure. Front. Neurol..

[12] Risling M (2011). Mechanisms of blast induced brain injuries, experimental studies in rats. NeuroImage..

[13] Feng K (2016). Biomechanical responses of the brain in swine subject to free-field blasts. Front. Neurol..

[14] Konan L (2019). Multi-focal neuronal ultrastructural abnormalities and synaptic alterations in mice after low-intensity blast exposure. J. Neurotrauma..

[15] Lu J (2012). Effect of blast exposure on the brain structure and cognition in *macaca fascicularis*. J. Neurotrauma..

[16] Pun P (2011). Low level primary blast injury in rodent brain. Front. Neurol..

[17] Rubovitch V (2011). A mouse model of blast-induced mild traumatic brain injury. Exp. Neurol..

[18] Song H (2018). Ultrastructural brain abnormalities and associated behavioral changes in mice after low-intensity blast exposure. Behav. Brain. Res..

[19] Ahmed F (2010). Time-dependent changes of protein biomarker levels in the cerebrospinal fluid after blast traumatic brain injury. Electrophoresis.

[20] Bauman R (2009). An introductory characterization of a combat-casualty-care relevant swine model of closed head injury resulting from exposure to explosive blast. J. Neurotrauma..

[21] De Lanerolle N (2011). Characteristics of an explosive blast-induced brain injury in an experimental model. J. Neuropathol. Exp. Neurol..

[22] Gyorgy A (2011). Time-dependent changes in serum biomarker levels after blast traumatic brain injury. J. Neurotrauma..

[23] Huang E (2013). Repeated blast exposure alters open field behavior recorded under low illumination. Brain Res..

[24] Verma S (2015). Multi-echo susceptibility-weighted imaging and histology of open-field blast induced traumatic brain injury in a rat model. NMR Biomed..

[25] DeGasperi R (2012). Acute blast injury reduces brain abeta in two rodent species. Front. Neurol..

[26] Baalman K, Cotton R, Rasband S, Rasband M (2013). Blast wave exposure impairs memory and decreases axon initial segment length. J. Neurotrauma..

[27] Gama Sosa M (2017). Lack of chronic neuroinflammation in the absence of focal hemorrhage in a rat model of low-energy blast-induced TBI. Acta Neuropathol. Commun..

[28] Gama Sosa M (2019). Low-level blast exposure disrupts gliovascular and neurovascular connections and induces a chronic vascular pathology in rat brain. Acta Neuropathol. Commun..

[29] Garman R (2011). Blast exposure in rats with body shielding is characterized primarily by diffuse axonal injury. J. Neurotrauma..

[30] Goodrich J (2016). Neuronal and glial changes in the brain resulting from explosive blast in an experimental model. Acta Neuropathol. Comm..

[31] Robinson M (2019). Positron emission tomography of tau in Iraq and Afghanistan veterans with blast neurotrauma. NeuroImage Clin..

[32] Catafau A (2016). Cerebellar amyloid-βplaques: how frequent are they and do they influence 18F-Florbetaben SVU ratios. J. Nucl. Med..

[33] Jacobs H (2018). The cerebellum in Alzheimer’s disease: evaluating its role in cognitive decline. Brain.

[34] Arun P (2013). Distinct patterns of expression of traumatic brain injury biomarkers after blast exposure: role of compromised cell membrane integrity. Neurosci. Lett..

[35] Chen M (2018). Proteomic profiling of mouse brains exposed to blast induced mild traumatic brain injury reveals changes in axonal proteins and phosphorylated tau. J. Alzheimer Dis..

[36] Huber B (2013). Blast exposure causes early and persistent aberrant phospho- and cleaved-tau expression in a murine model of mild blast-induced traumatic brain injury. J. Alzheimer Dis..

[37] Perez-Polo J (2015). A rodent model of mild traumatic brain blast injury. J. Neurosci. Res..

[38] Wang Y (2017). Primary Blast-Induced changes in Akt and GSK3β Phosphorylation in Rat Hippocampus. Front. Neurol..

[39] Lucke-Wold B (2016). Endoplasmic reticulum stress implicated in chronic traumatic encephalopathy. J. Neurosurg..

[40] Zhao S, Fu J, Liu X, Wang T, Zhang J, Zhao Y (2012). Activation of Akt/GSK-3beta/beta-catenin signaling pathway is involved in survival of neurons after traumatic brain injury in rats. Neurol Res..

[41] Feng Q, Cheng B, Yang R, Sun F, Zhu C (2005). Dynamic changes of phosphorylated tau in mouse hippocampus after cold water stress. Neurosci. Lett..

[42] Okawa Y, Ishiguro K, Fujita S (2003). Stress-induced hyperphosphorylation of tau in the mouse brain. FEBS Lett..

[43] Yanagisawa M, Planel E, Ishiguro K, Fujita S (1999). Starvation induces tau hyperphosphorylation in mouse brain: implications for Alzheimer's disease. FEBS Lett..

[44] Stuhmiller, J. Modeling of the Non-Auditory Response to Blast Overpressure: Summary of Blast Overpressure Field Data. Ft. Detrick (MD): U.S. Army Medical Research and Development Command; 1990 Jan, Document Number: DAMD17–85-C-5238. [accessed April 26, 2019].https://apps.dtic.mil/dtic/tr/fulltext/u2/a223667.pdf.

[45] Engel, C., Hoch, E., Simmons, M. The Neurological Effects of Repeated Exposure to Military Occupational Blast: Implications for Prevention and Health: Proceedings, Findings, and Expert Recommendations from the Seventh Department of Defense State-of-the-Science Meeting, Santa Monica (CA): RAND Corporation, Army Research Division; 2019, Document Number: CF-380/1-A [Accessed 2019 Oct 16]. https://www.rand.org/pubs/conf_proceedings/CF380z1.html

[46] Kirsch J, Klaus J, Blizzard K, Hurn P, Murphy S (2002). Pain evaluation and response to buprenorphine in rats subjected to sham middle cerebral artery occlusion. Contemp. Top. Lab. Anim. Sci..

[47] Shapira Y (1988). Experimental closed head injury in rats: mechanical, pathophysiologic, and neurologic properties. Crit. Care Med..

[48] Bederson J (1986). Rat middle cerebral artery occlusion: evaluation of the model and development of a neurologic examination. Stroke.

[49] Tupper D, Wallace R (1980). Utility of the neurological examination in rats. Acta Neurobiol. Exp..

[50] Gensel J (2006). Behavioral and histological characterization of unilateral cervical spinal cord contusion injury in rats. J. Neurotrauma..

[51] Hamers F, Lankhorst A, van Laar T, Veldhuis W, Gispen W (2001). Automated quantitative gait analysis during overground locomotion in the rat: its application to spinal cord contusion and transection injuries. J. Neurotrauma..

[52] Neumann M (2008). Assessing gait impairment following experimental traumatic brain injury in mice. J. Neurosci. Methods..

[53] Paxinos G, Watson C (2014). Paxinos and Watson's: The rat brain in stereotaxic coordinates.

[54] Buée L, Bussière T, Buée-Scherrer V, Delacourte A, Hof P (2000). Tau protein isoforms, phosphorylation and role in neurodegenerative disorders. Brain Res. Brain Res. Rev..

[55] Ishiguro K (1993). Glycogen synthase kinase 3 beta is identical to tau protein kinase I generating several epitopes of paired helical filaments. FEBS Lett..

[56] Cross D, Alessi D, Cohen P, Andjelkovich M, Hemmings B (1995). Inhibition of glycogen synthase kinase-3 by insulin mediated by protein kinase B. Nature.

[57] Doble B, Woodgett J (2003). GSK-3: tricks of the trade for a multi-tasking kinase. J. Cell Sci..

[58] Qian W (2010). PP2A regulates tau phosphorylation directly and also indirectly via activating GSK-3beta. J. Alzheimers Dis..

[59] Sontag E, Nunbhakdi-Craig V, Lee G, Bloom G, Mumby M (1996). Regulation of the phosphorylation state and microtubule-binding activity of Tau by protein phosphatase 2A. Neuron.

[60] Hatten M, Liem R, Shelanski M, Mason C (1991). Astroglia in CNS injury. Glia..

[61] Donat C, Scott G, Gentleman S, Sastre M (2017). Microglial activation in traumatic brain injury. Front. Aging Neurosci..

[62] Hernandez A (2018). Exposure to mild blast forces induces neuropathological deficits and biochemical changes. Mol. Brain..

[63] Park E, Gottleib J, Cheung B, Shek P, Baker A (2011). A model of low-level primary blast brain trauma results in cytoskeletal proteolysis and chronic functional impairment in the absence of lung barotrauma. J. Neurotrauma..

[64] Pike B (2001). Accumulation of non-erythroid alpha II-spectrin and calpain-cleaved alpha II-spectrin breakdown products in cerebrospinal fluid after traumatic brain injury in rats. J. Neurochem..

[65] Pike B (2004). Accumulation of calpain and caspase-3 proteolytic fragments of brain-derived alpha II-spectrin in cerebrospinal fluid after middle cerebral artery occlusion in rats. J. Cereb. Blood Flow Metab..

[66] Valiyaveettil M (2014). Cytoskeletal protein α-II spectrin degradation in the brain of repeated blast exposed mice. Brain Res..

[67] Du X (2013). Effects of antioxidant treatment on blast-induced brain injury. PLoS ONE.

[68] Gentleman S, Nash M, Sweeting C, Graham D, Roberts G (1993). *B–*Amyloid precursor protein (B*–*APP) as a marker for axonal injury after head injury. Neurosci. Lett..

[69] Kuehn R (2011). Rodent model of direct cranial blast injury. J. Neurotrauma..

[70] Pierce J, Trojanowski J, Graham D, Smith D, McIntosh T (1996). Immunohistochemical characterization of alterations in the distribution of amyloid precursor proteins and *B*-amyloid peptide after experimental brain injury in the rat. J. Neurosci..

[71] Sheriff F, Bridges L, Sivaloganathan S (1994). Early detection of axonal injury after human head trauma using immunocytochemistry for *B*-amyloid precursor protein. Acta Neuropathol..

[72] Van Den Heuvel C (1999). Upregulation of amyloid precursor protein messenger RNA in response to traumatic brain injury: an ovine head impact model. Exp. Neurol..

[73] Wang H (2018). Novel-graded traumatic brain injury model in rats induced by closed head impacts. Neuropathology..

