# Supplementary figures and images for: Explosive-driven double-blast exposure: molecular, histopathological, and behavioral consequences
Source: Sci Rep. 2020 Oct 15;10:17446. doi: 10.1038/s41598-020-74296-2 (PMC7566442; doi:10.1038/s41598-020-74296-2)

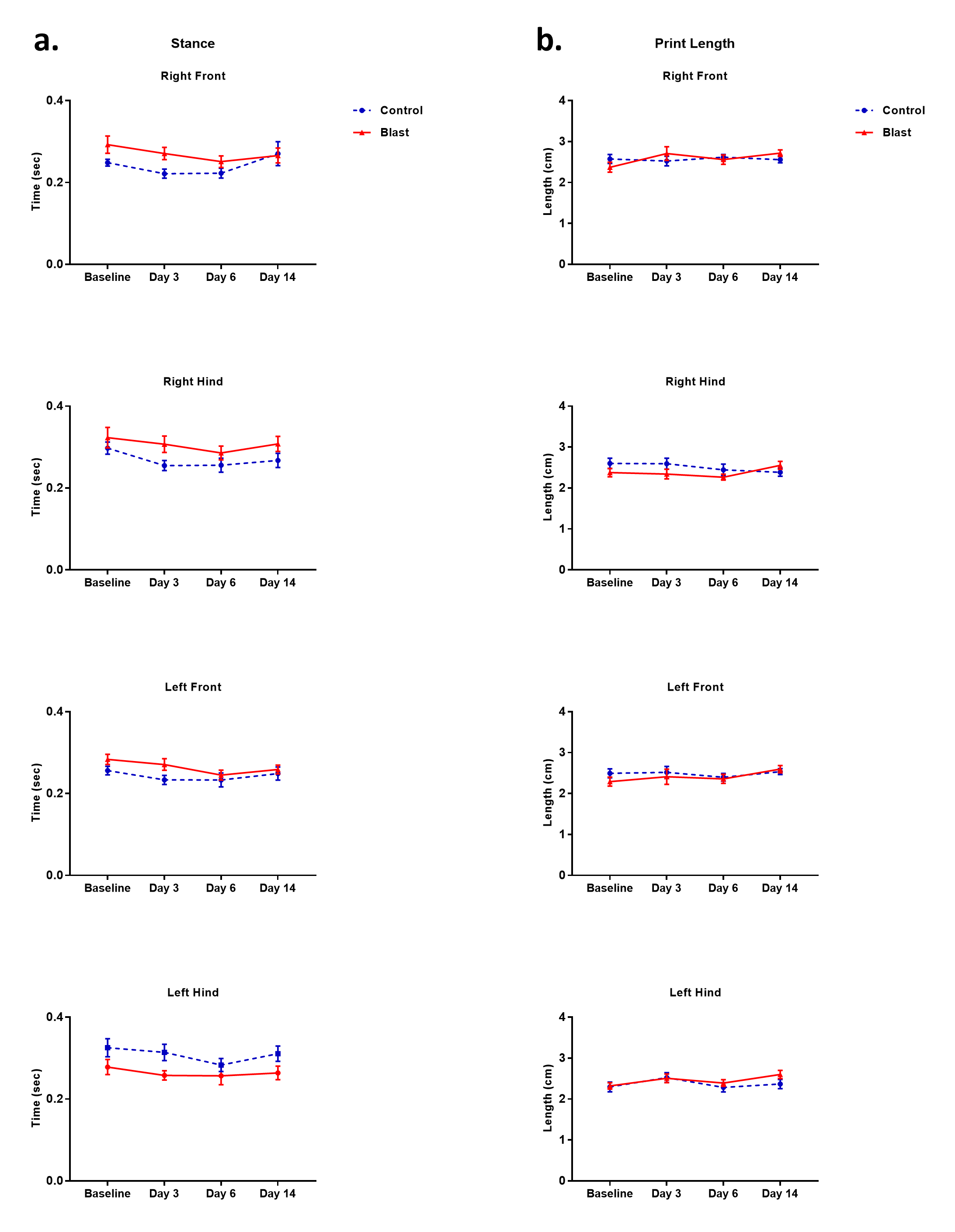

Supplement: Supplementary file 1 — Supplementary Figure 1. [file 41598_2020_74296_MOESM1_ESM.tif]

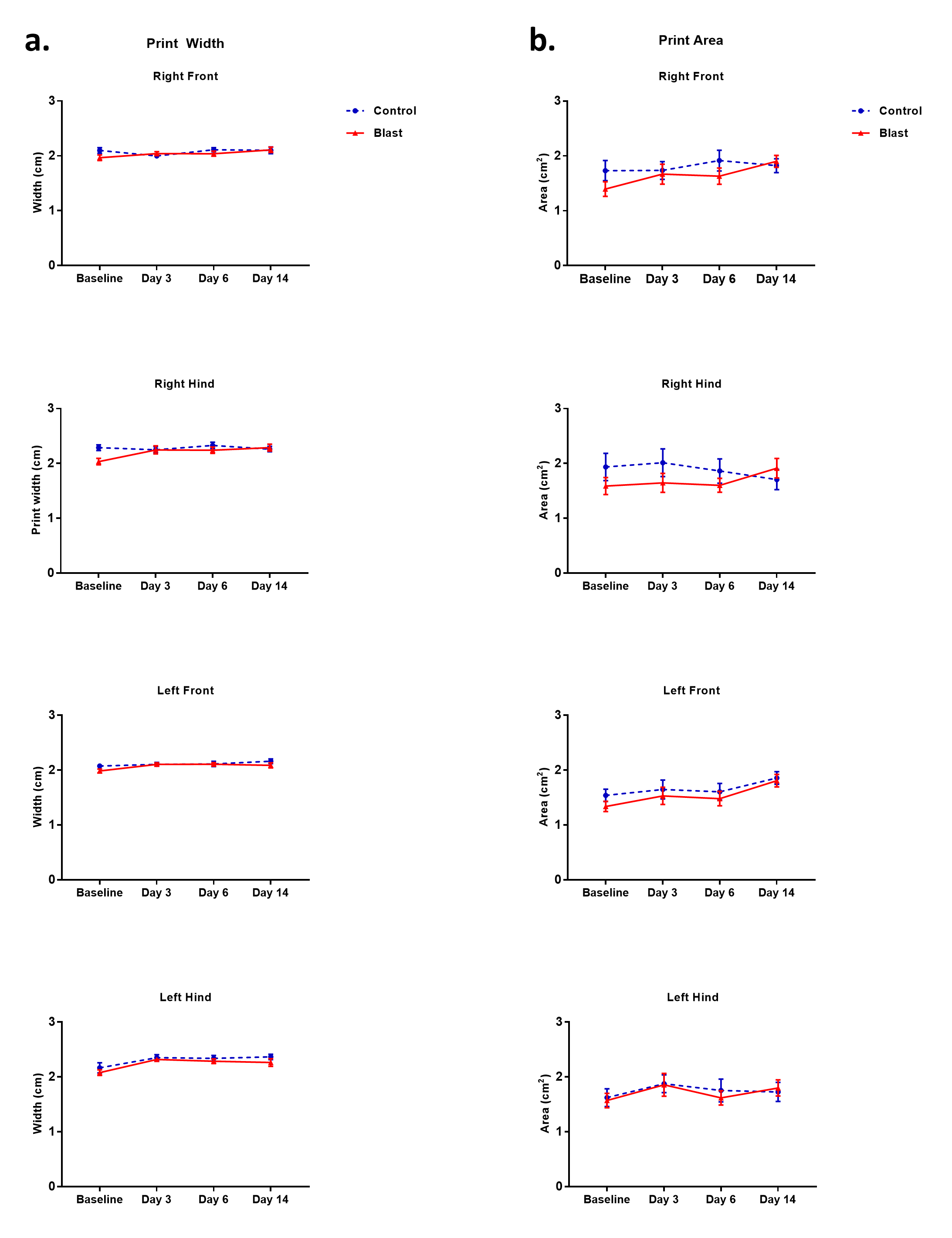

Supplement: Supplementary file 2 — Supplementary Figure 2. [file 41598_2020_74296_MOESM2_ESM.tif]

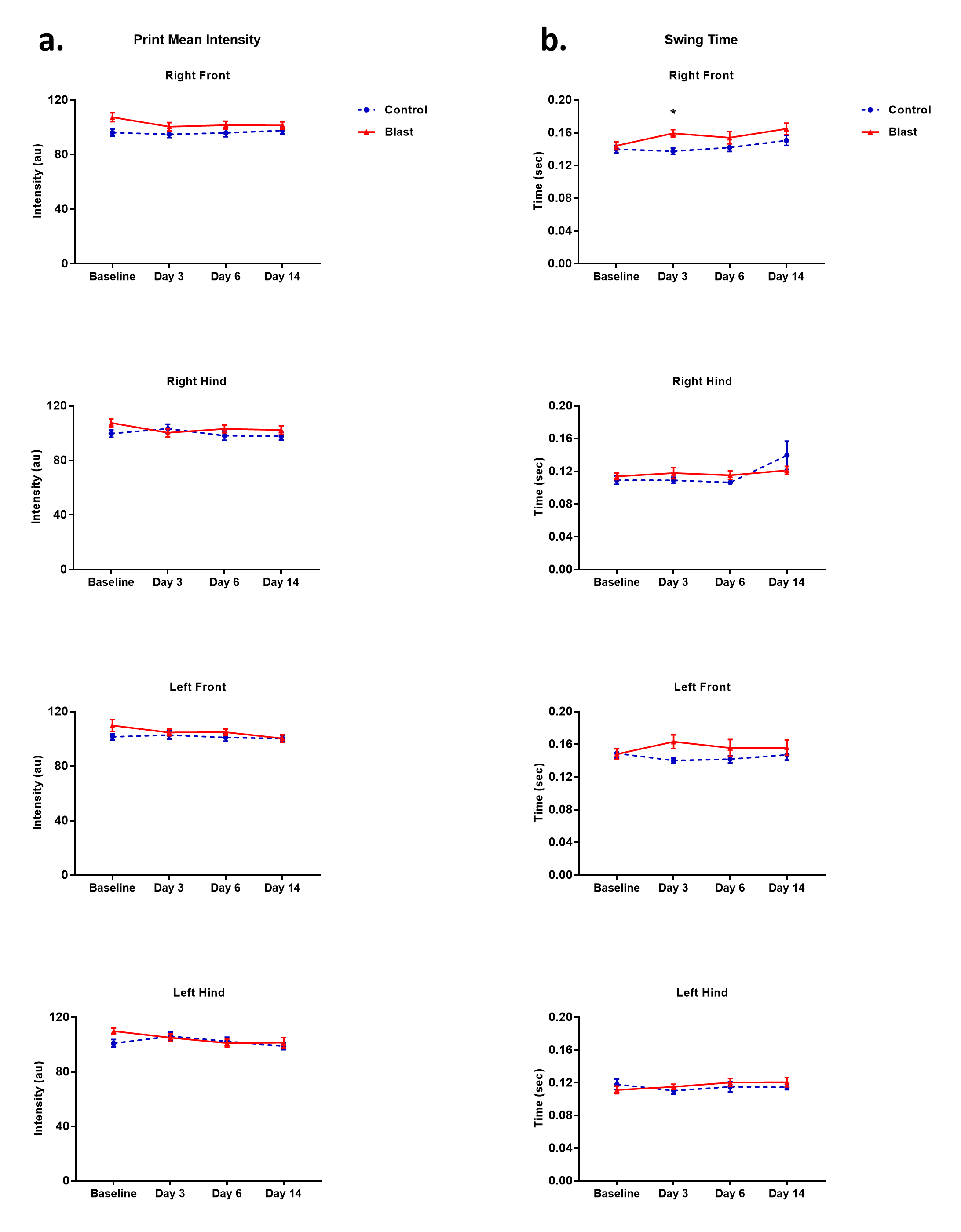

Supplement: Supplementary file 3 — Supplementary Figure 3. [file 41598_2020_74296_MOESM3_ESM.tif]

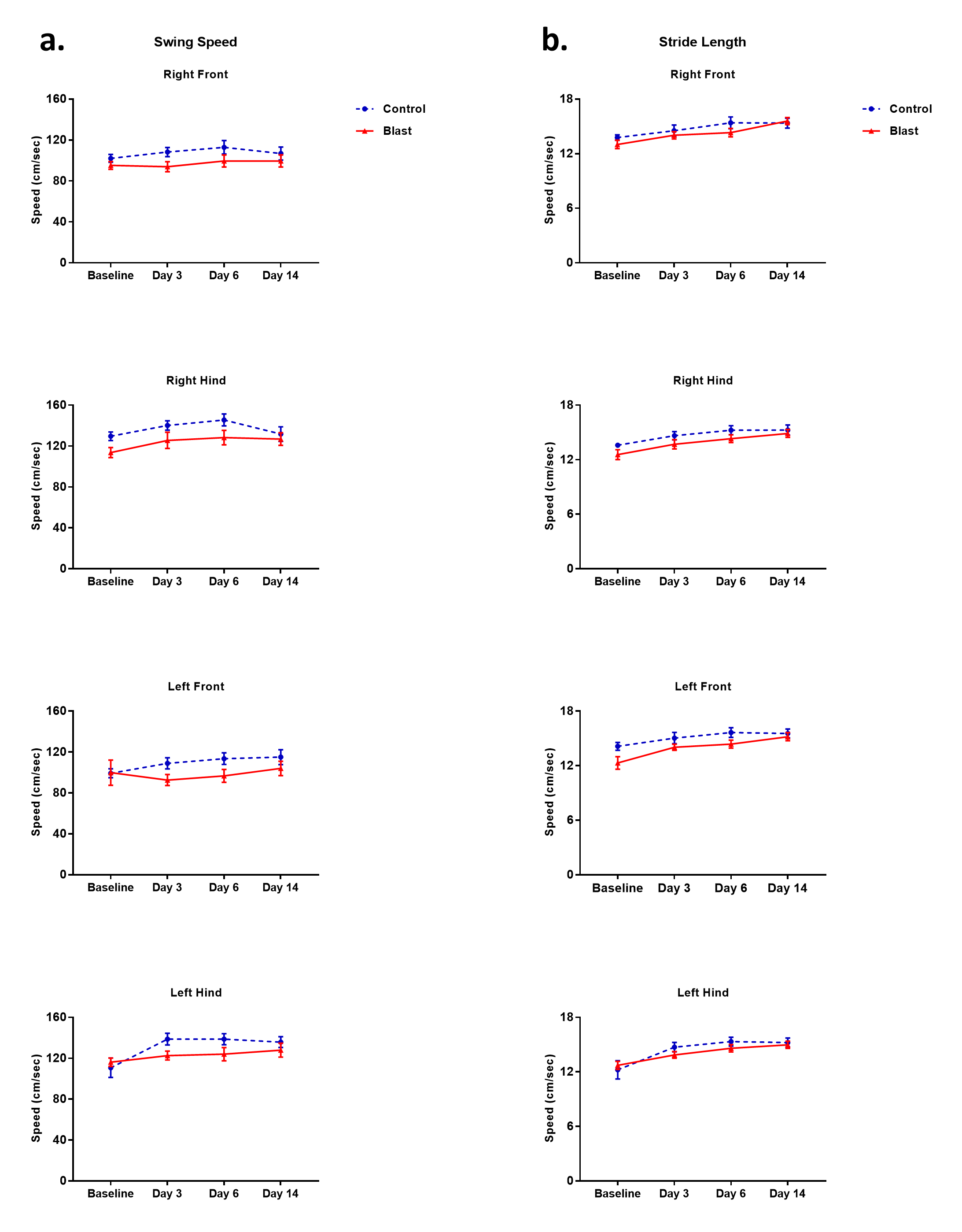

Supplement: Supplementary file 4 — Supplementary Figure 4. [file 41598_2020_74296_MOESM4_ESM.tif]

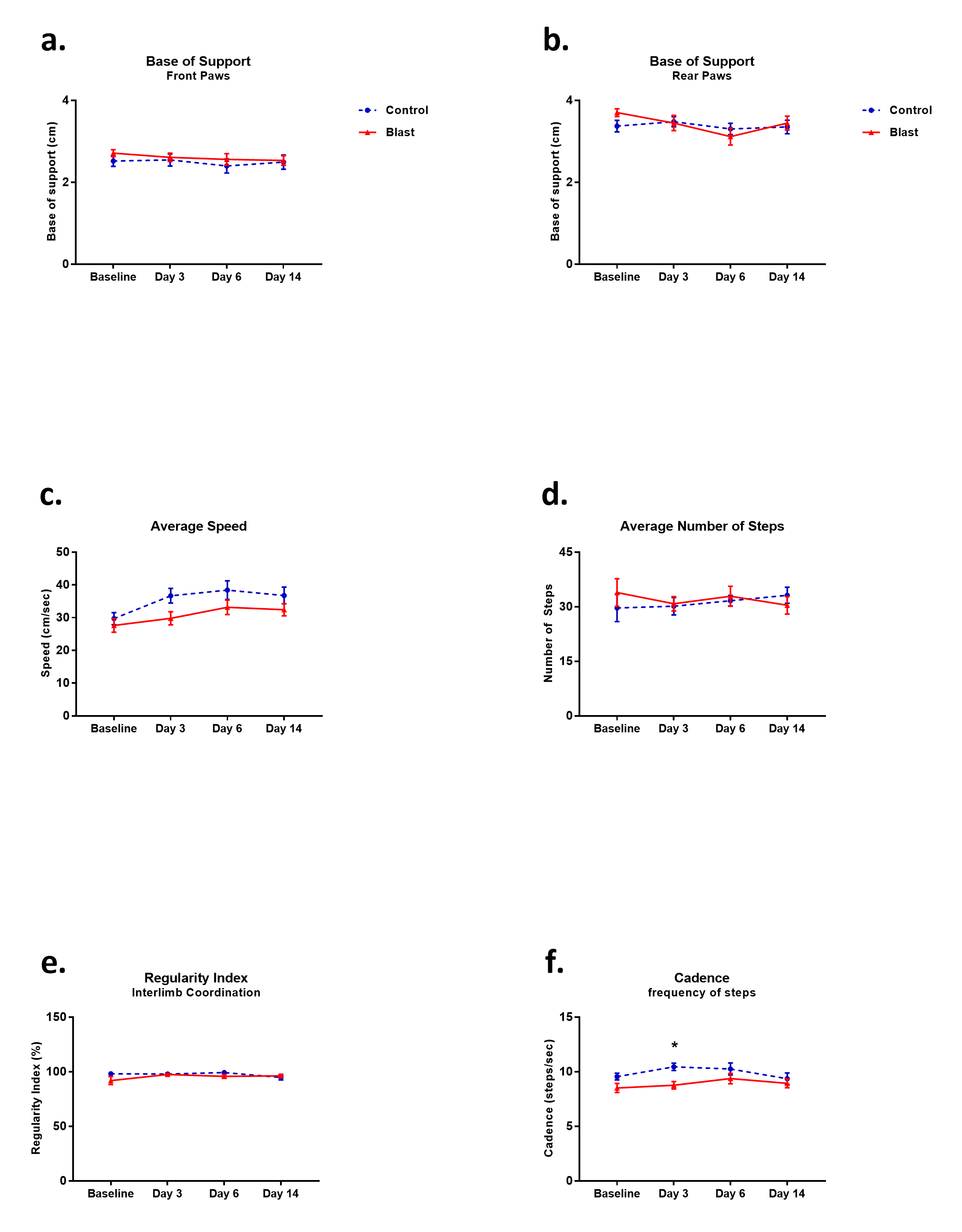

Supplement: Supplementary file 5 — Supplementary Figure 5. [file 41598_2020_74296_MOESM5_ESM.tif]

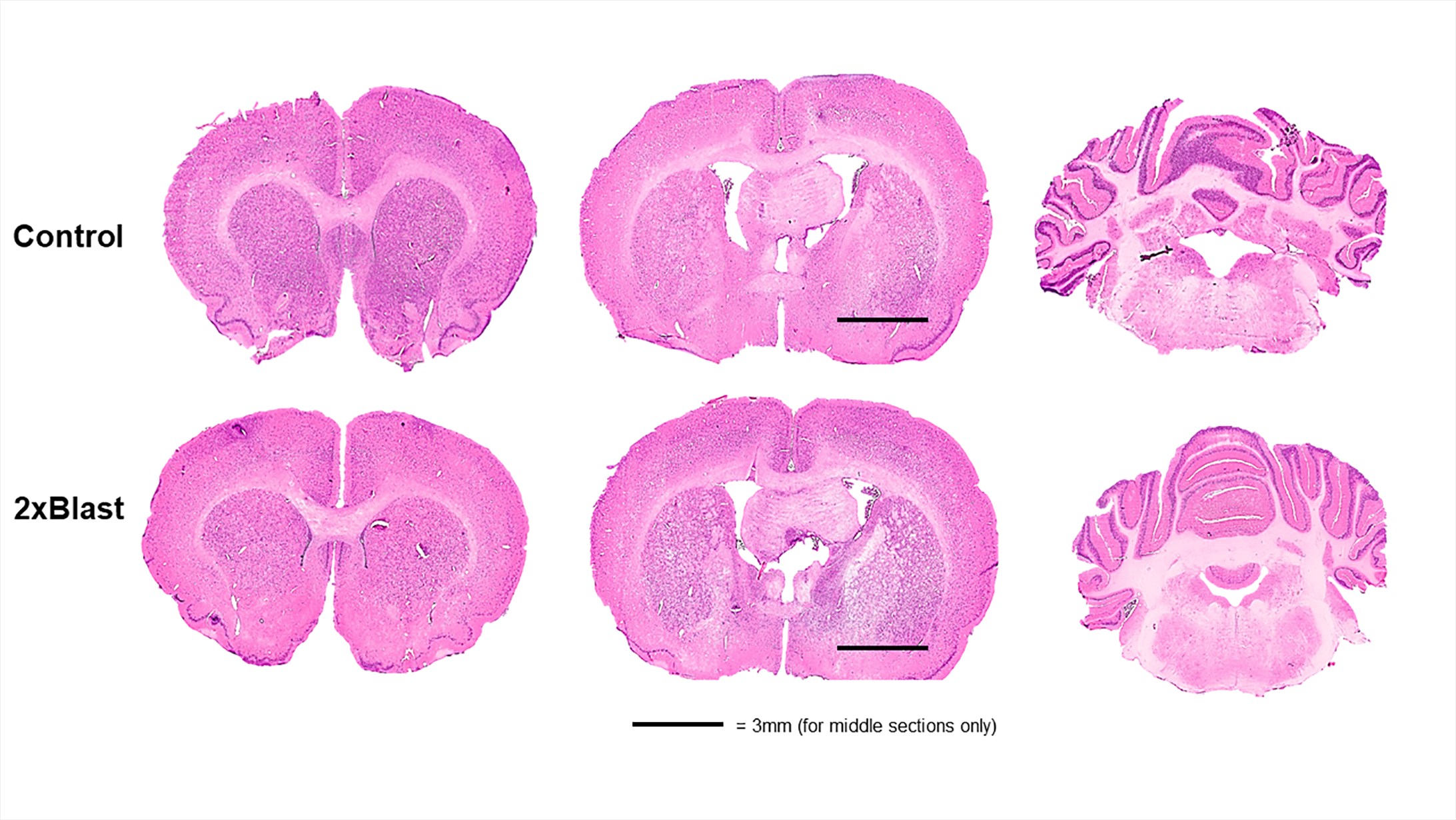

Supplement: Supplementary file 6 — Supplementary Figure 6. [file 41598_2020_74296_MOESM6_ESM.tif]

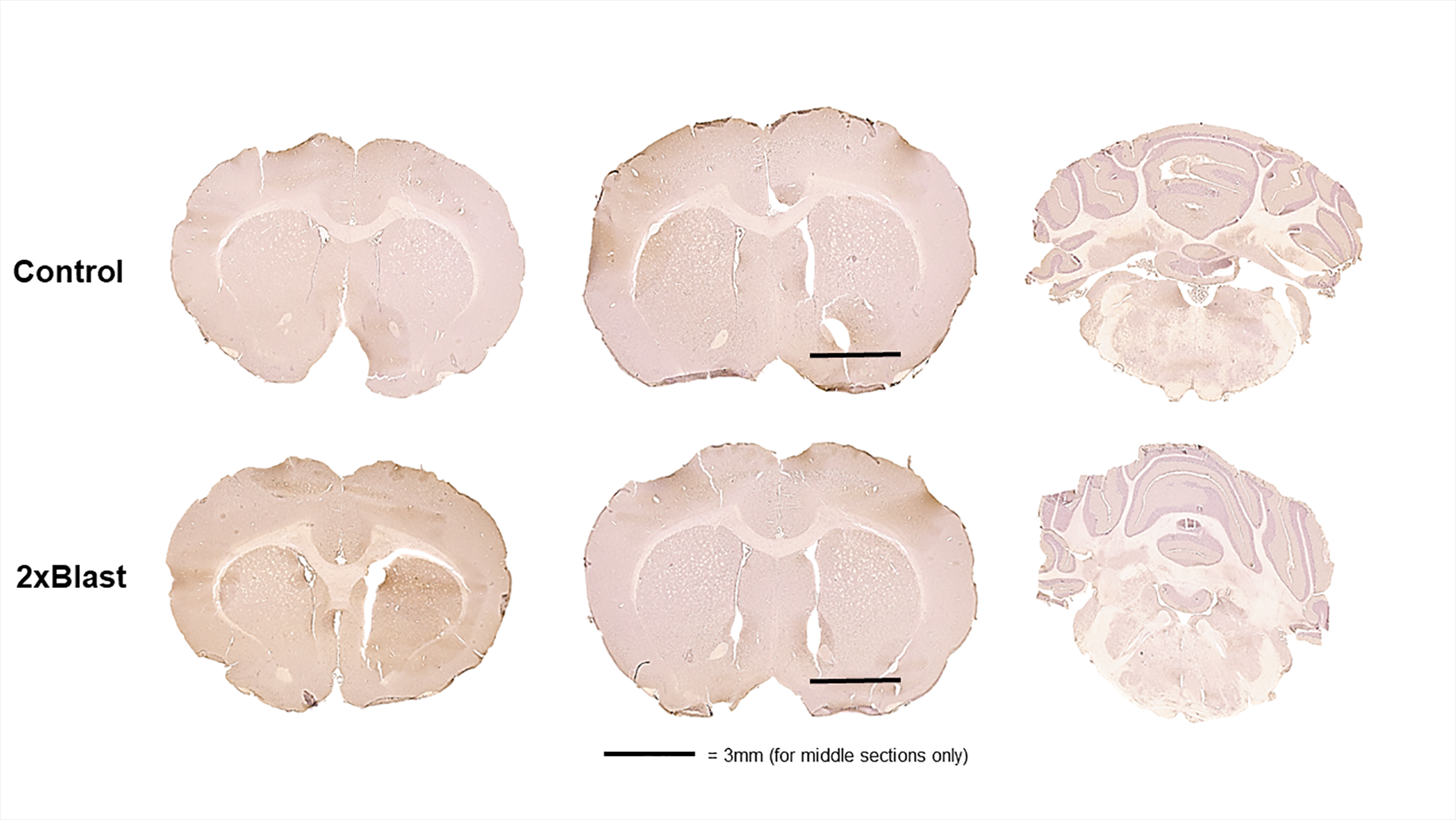

Supplement: Supplementary file 7 — Supplementary Figure 7. [file 41598_2020_74296_MOESM7_ESM.tif]

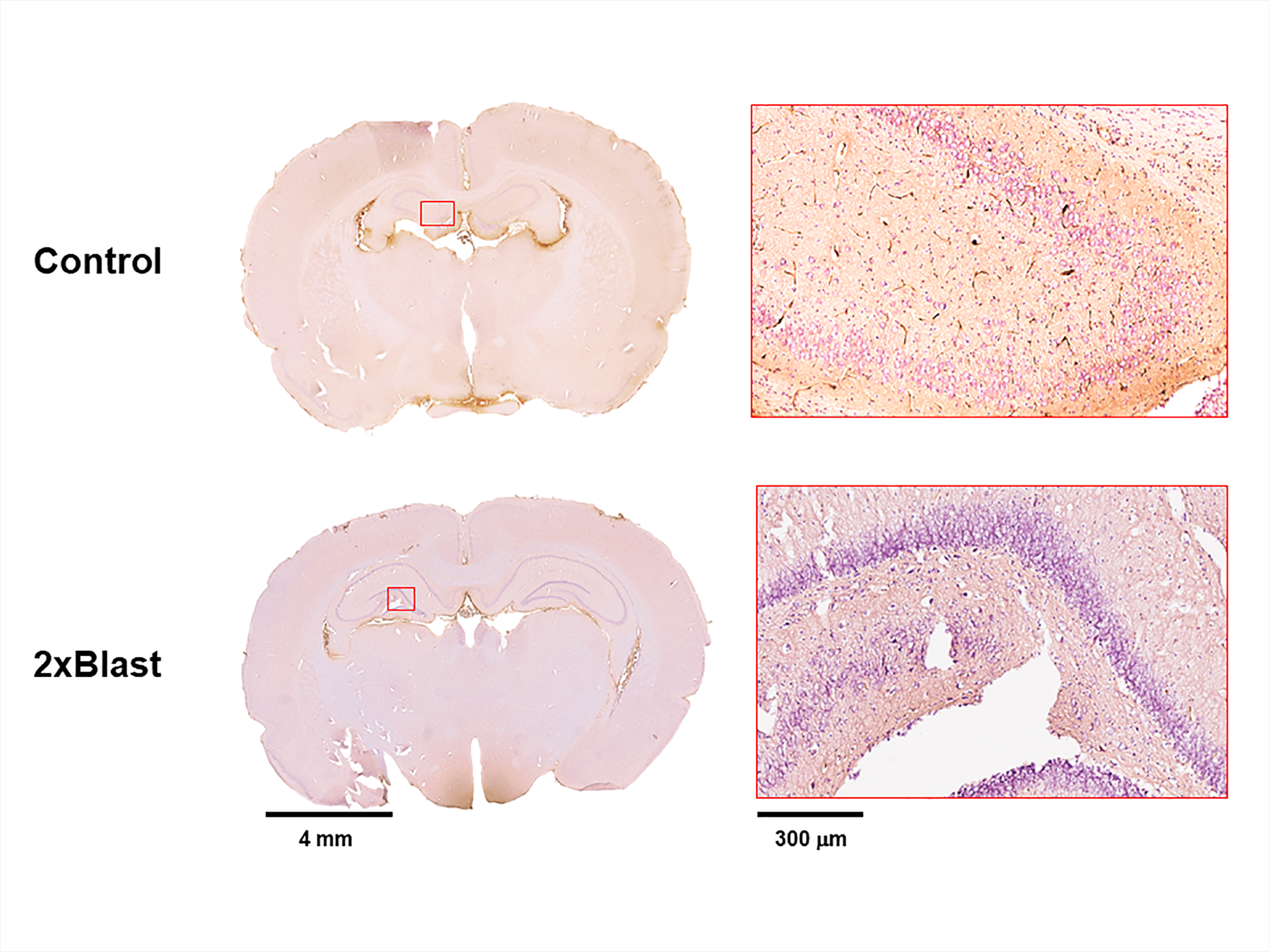

Supplement: Supplementary file 8 — Supplementary Figure 8. [file 41598_2020_74296_MOESM8_ESM.tif]

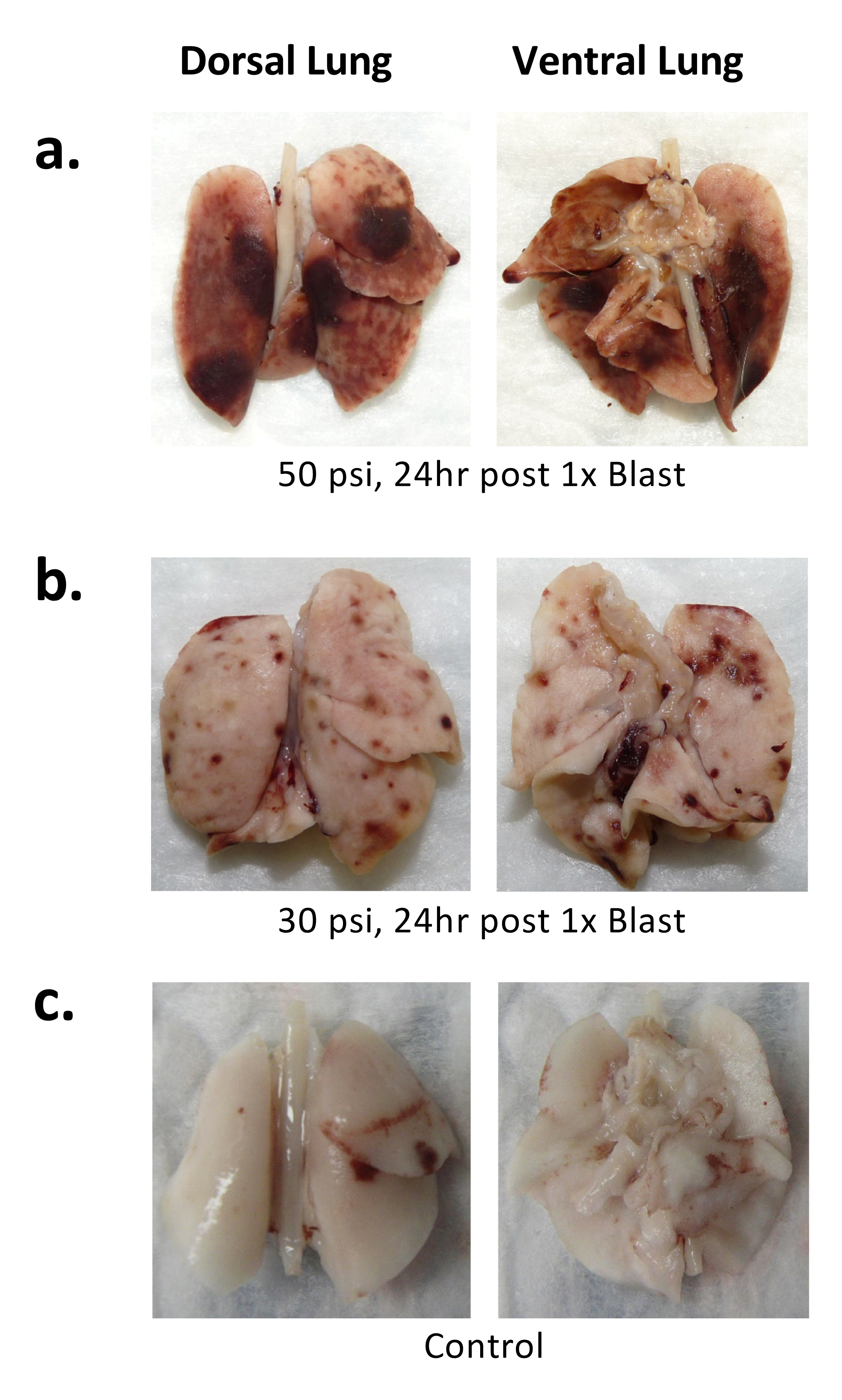

Supplement: Supplementary file 9 — Supplementary Figure 9. [file 41598_2020_74296_MOESM9_ESM.tif]

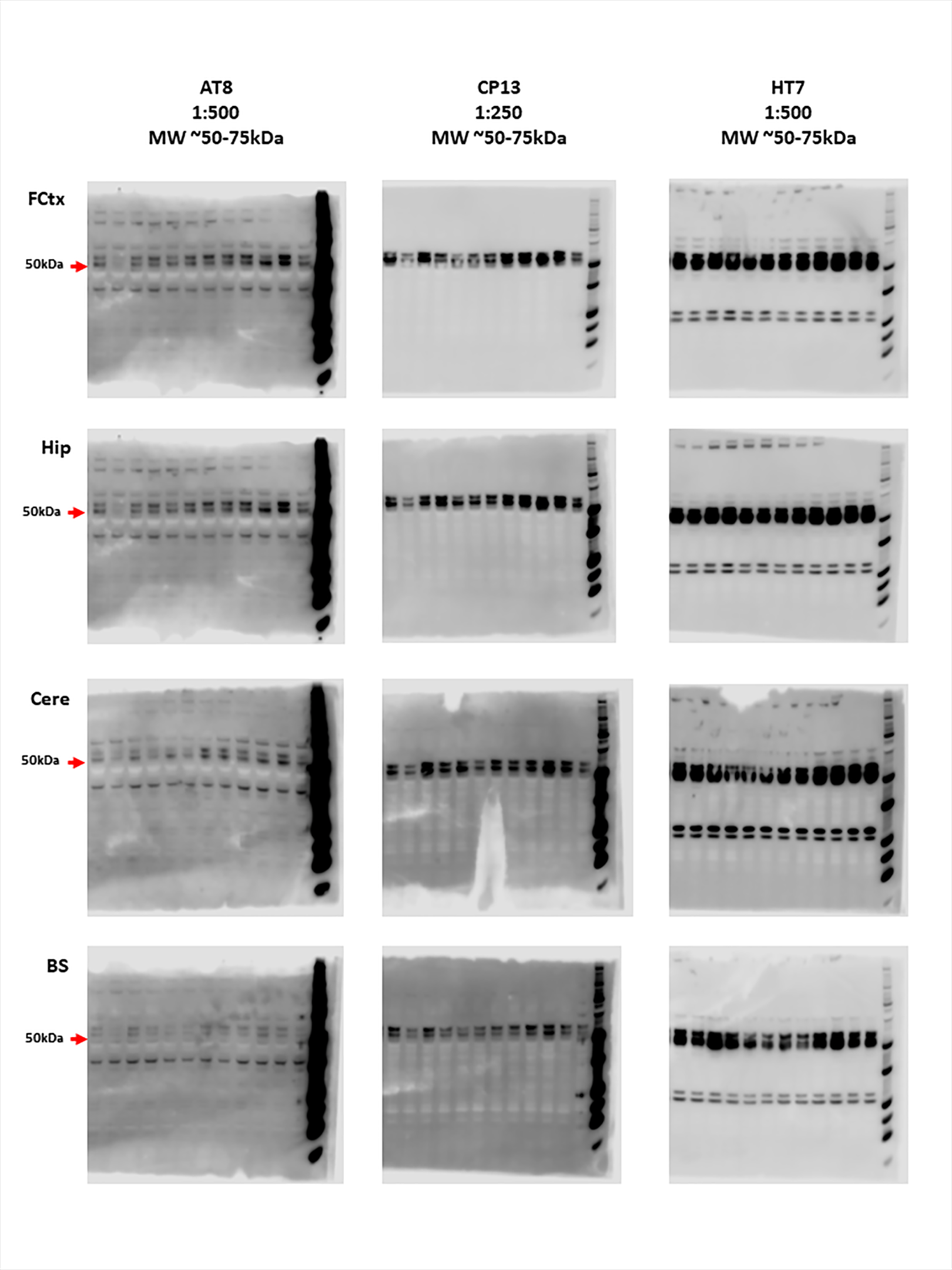

Supplement: Supplementary file 10 — Supplementary Figure 10. [file 41598_2020_74296_MOESM10_ESM.tif]

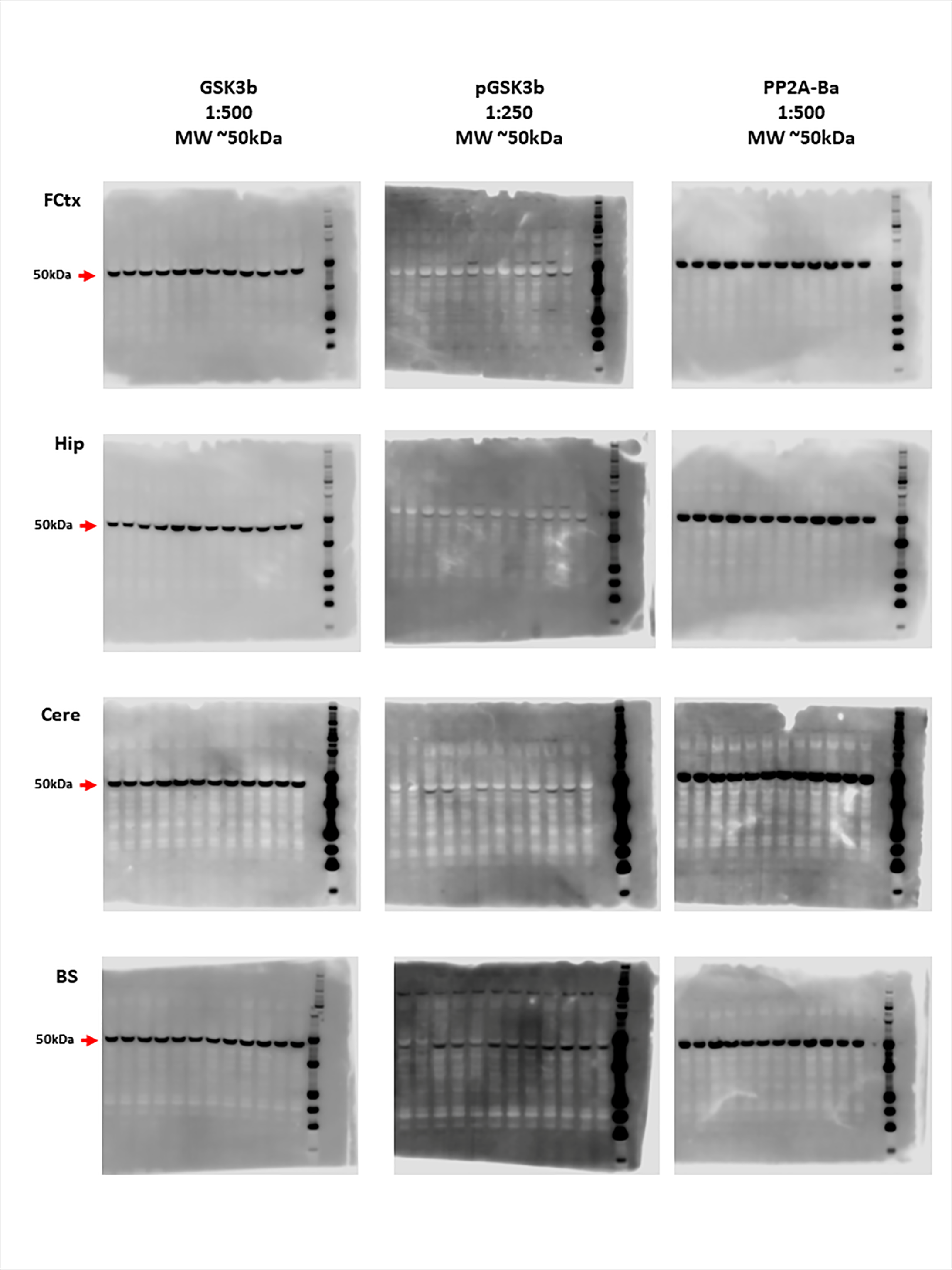

Supplement: Supplementary file 11 — Supplementary Figure 11. [file 41598_2020_74296_MOESM11_ESM.tif]

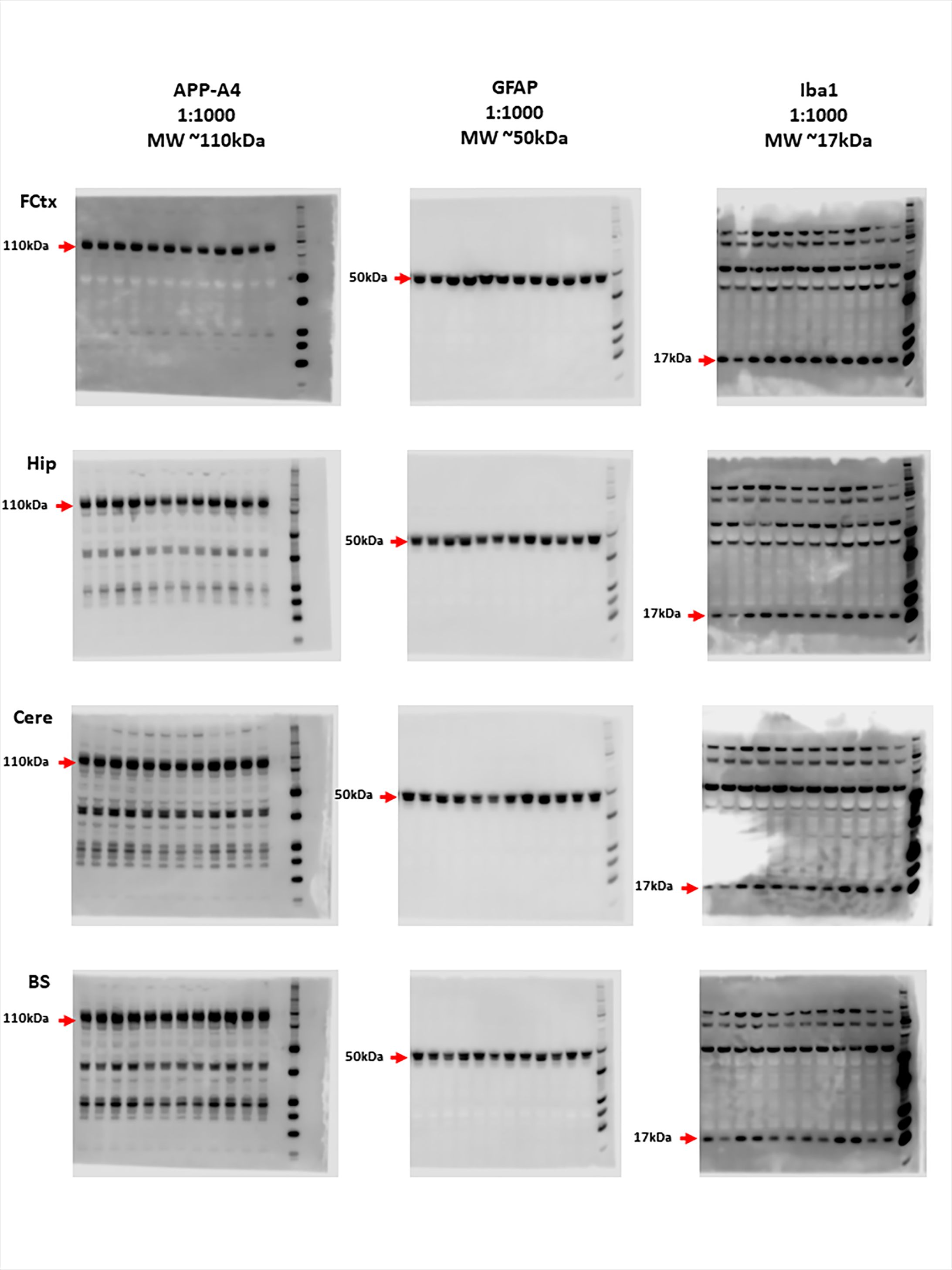

Supplement: Supplementary file 12 — Supplementary Figure 12. [file 41598_2020_74296_MOESM12_ESM.tif]

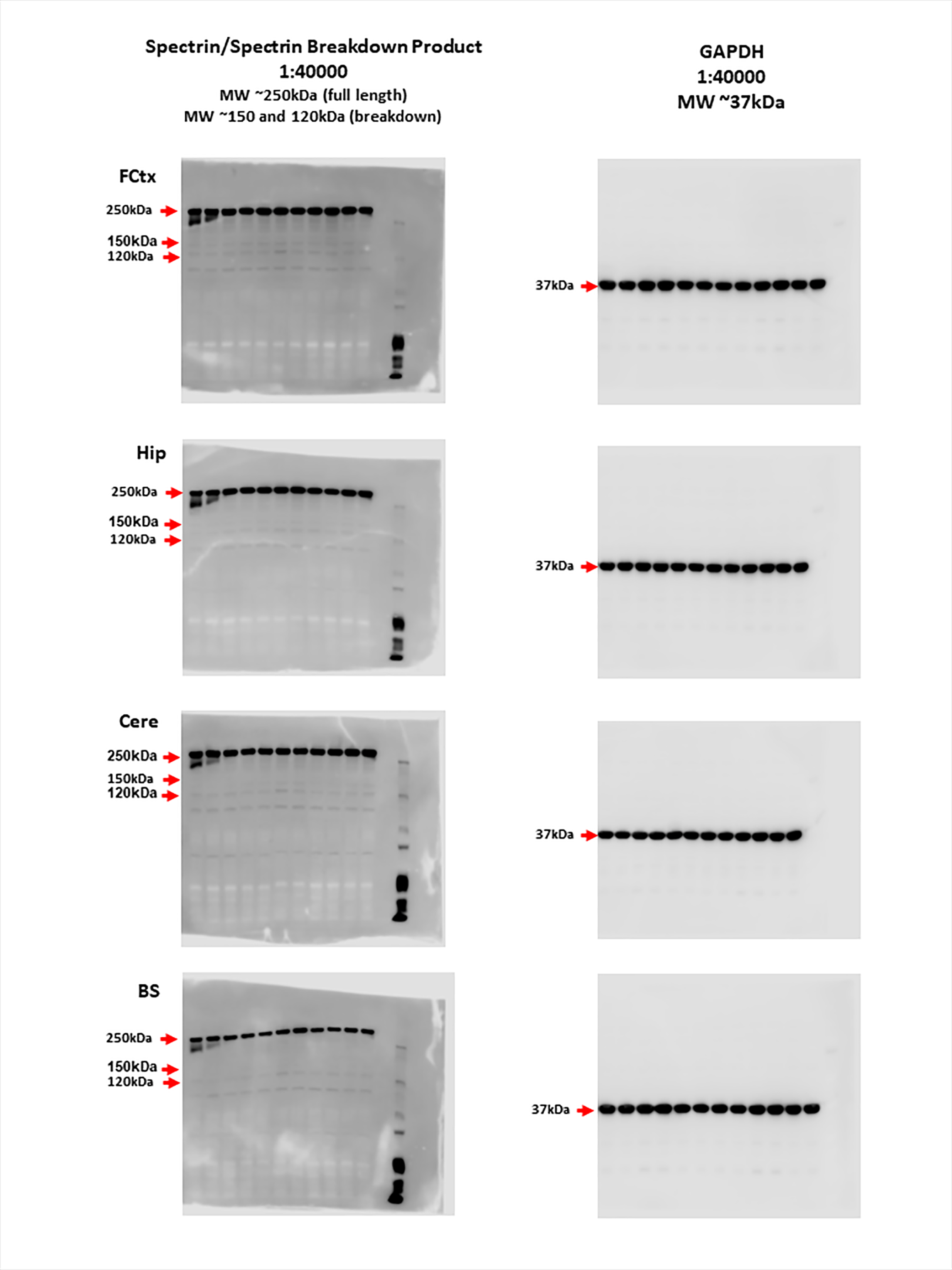

Supplement: Supplementary file 13 — Supplementary Figure 13. [file 41598_2020_74296_MOESM13_ESM.tif]
