# Supplementary material for: Explosive-driven double-blast exposure: molecular, histopathological, and behavioral consequences
Source: Sci Rep. 2020 Oct 15;10:17446. doi: 10.1038/s41598-020-74296-2 (PMC7566442; doi:10.1038/s41598-020-74296-2)
Supplement: Supplementary file 14 — Supplementary Legends. [file 41598_2020_74296_MOESM14_ESM.docx]

**Supplementary Figure S1**

Gait Outcomes Following Double Blast Exposure: No significant differences between 2xB and Ctl in the parameters of Stance **(A.)** or Print Length **(B.)** over the course of the study as analyzed by 2-way repeated measures ANOVA. Each parameter is analyzed separately for each of the 4 paws, n=12 per group.

**Supplementary Figure S2**

Gait Outcomes Following Double Blast Exposure: No significant differences between 2xB and Ctl in the parameters of **Print Width** **(A.)** or **Print Area** **(B.)** over the course of the study as analyzed by 2-way repeated measures ANOVA. Each parameter is analyzed separately for each of the 4 paws, n=12 per group.

**Supplementary Figure S3**

Gait Outcomes Following Double Blast Exposure: No significant differences between 2xB and Ctl in the parameters of **Print Mean Intensity** **(A.)** over the course of the study as analyzed by 2-way repeated measures ANOVA. At Day 3 following double blast exposure, there is a significant difference between 2xB and Ctl in the measure of **Swing Time (B.)** in the right front paw only (*p*=0.03) as analyzed by 2-way repeated measures ANOVA. No other paws demonstrated a significant difference on this parameter and the difference is gone by Day 6. Each parameter is analyzed separately for each of the 4 paws, n=12 per group.

**Supplementary Figure S4**

Gait Outcomes Following Double Blast Exposure: Gait Outcomes Following Double Blast Exposure: No significant differences between 2xB and Ctl in the parameters of **Swing Speed** **(A.)** or **Stride Length** **(B.)** over the course of the study as analyzed by 2-way repeated measures ANOVA. Each parameter is analyzed separately for each of the 4 paws, n=12 per group.

**Supplementary Figure S5**

Gait Outcomes Following Double Blast Exposure: No significant differences between 2xB and Ctl in the parameters of **Base of Support (Front and Rear)** **(A-B)**, **Average Speed** **(C.)**, **Average Number of Steps** **(D.)** or **Regularity Index** **(E.)** (aka-interlimb coordination) over the course of the study as analyzed by 2-way repeated measures ANOVA. At Day 3 following double blast exposure, there is a significant difference between 2xB and Ctl in the measure of **Cadence** **(F.)** (aka- frequency of steps) (*p*=0.03) as analyzed by 2-way repeated measures ANOVA. This difference is not present at any other time point measured. For these specific gait parameters, paws are not analyzed separately, n=12 per group.

**Supplementary Figure S6**

H&E Stain on Control and Double Blast Exposed Brain: No evidence of gross histopathologic changes across three representative brain regions in control versus double blast exposed brains. Slides were scanned at 20x on the Aperio AT2 slide scanner and images evaluated in Aperio ImageScope software. Scalebar = 3mm (for middle sections only).

**Supplementary Figure S7**

APP Immunohistochemistry on Control and Double Blast Exposed Brain: No evidence of APP immunopositive staining across three representative brain regions in control versus double blast exposed brains. Slides were scanned at 20x on the Aperio AT2 slide scanner and images evaluated in Aperio ImageScope software. Scalebar = 3mm (for middle sections only).

**Supplementary Figure S8**

AT8 Immunohistochemistry on Control and Double Blast Exposed Brain: No evidence of AT8 immunopositive staining in the hippocampus in control versus double blast exposed brains. Slides were scanned at 20x on the Aperio AT2 slide scanner and images evaluated in Aperio ImageScope software. Scalebar = 4mm (for 20x) or 300μm (for 100x).

**Supplementary Figure S9**

Lung Damage Assessment at Varying Blast Intensities: Gross images of rat lungs following exposure to a single blast wave at **(A.)** 48 psi (330 kPa) and **(B.)** 28 psi (193 kPa) as compared to **(C.)** control lung. All lung samples assessed 24 hours following blast exposure or control procedure.

**Supplementary Figure S10**

Images of the full-length blots for AT8, CP13 and HT7 in all 4 brain regions of interest.

**Supplementary Figure S11**

Images of the full-length blots for GSK3β, pGSK3β and PP2A-Bα in in all 4 brain regions of interest.

**Supplementary Figure S12**

Images of the full-length blots for APP-A4, GFAP and Iba1 in all 4 brain regions of interest.

**Supplementary Figure S13**

Images of the full-length blots for Spectrin/Spectrin Breakdown Products and a representative series of GAPDH in all 4 brain regions of interest. All blots were stripped one time and reprobed for GAPDH for normalization in densitometric analyses. This GAPDH series comes from the strip of Iba1 blots.
